# miR-184, a downregulated ovary-elevated miRNA transcriptionally activated by SREBF2, exerts anti-apoptotic properties in ovarian granulosa cells through inducing *SMAD3* expression

**DOI:** 10.1038/s41419-024-07286-1

**Published:** 2024-12-18

**Authors:** Baosen Shan, Yangan Huo, Zhennan Guo, Qiqi Li, Zengxiang Pan, Qifa Li, Xing Du

**Affiliations:** 1 College of Animal Science and Technology, Nanjing Agricultural University, Nanjing, 210095 China; 2 College of Animal Husbandry and Veterinary Medicine, Jiangsu Vocational College of Agriculture and Forestry, Zhenjiang, 212400 China

**Keywords:** Apoptosis, miRNAs

## Abstract

Follicular atresia is the primary threat to female fertility. miRNAs are dysregulated in granulosa cells (GCs) during follicular atresia, and have emerged as crucial regulators of the initiation and progression of follicular atresia. However, the downregulated ovary-elevated (OE) miRNAs and their biological functions in ovary remain elusive. Here, 13 downregulated OE miRNAs were systematically identified by integrating tissue expression high-throughput data and comparative transcriptome analyses, among which miR-184 was specifically highly expressed in ovary but dramatically downregulated during follicular atresia. Low miR-184 levels were also positively correlated with follicular atresia. Based on the in vitro GC and follicle culture system, we found that miR-184 suppressed GC apoptosis and follicular atresia. Mechanistically, miR-184 induced *SMAD3* transcription by acting as a saRNA, and also stabilized SMAD3 mRNA by directly binding to its 5′-UTR, which promoted TGF-β pathway activity and its anti-apoptotic effect. In addition, *miR-184* was transcribed independently of host gene, which was activated by SREBF2 in an H3K4me3-dependent manner. Comparative analysis revealed that SREBF2 expression and H3K4me3 enrichment on *miR-184* promoter in GCs from atretic follicles were dramatically reduced, which leads to the downregulation of miR-184 during follicular atresia. Moreover, the expression pattern, function, target, and regulatory mechanism of miR-184 among mammals are highly conserved and universal. Taken together, our findings demonstrate that miR-184, transcriptionally activated by SREBF2 in an H3K4me3-dependent manner, exerts anti-atretic effects by inducing *SMAD3* expression, highlighting that it is a promising regulator for improving follicular development, ovarian health and female fertility.

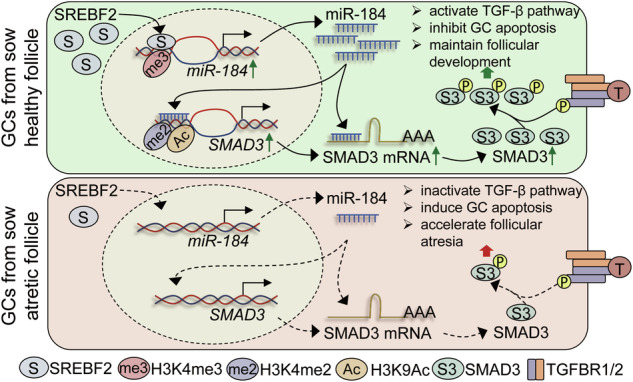

## Introduction

Nowadays, an increasing number of women are postponing their childbearing age with the accelerated life pace and increased work pressure [1]. However, their reproductive capacity declines after the age of 37, and rarely get pregnant after 45, due to ovarian aging and functional recession [2]. As the most important organ in female reproductive system, the primary functions of ovary are to support follicular development, ovulation, and hormone secretion [3]. Studies have found that 40% of infertile cases are caused by follicular development disorder and anovulation [4], indicating that follicles, the basic functional units of ovary, are essential for maintaining female fertility. Despite the huge storage in primordial follicle pool, physiological analysis reveals that most of the follicles undergo atresia and degeneration at each stage during development, with less than 1% being able to mature and ovulate [5], which is a selection process of dominant follicles, but resulting in the waste of biological resources. In addition, it was reported that severe atresia induced ovarian aging, diseases, and infertility [6]. Therefore, investigation of the underlying mechanism of follicular atresia is crucial for the advancement of reproductive medicine and livestock production.

Granulosa cells (GCs), a vital somatic cell type in follicles, adhere to inner membrane and surround the oocyte by forming a dense multilayered structure [7], which supports follicular development and induces oocyte maturation through the release of various steroid hormones and cytokines [8]. Clinical data and animal models revealed that the aberrant states (apoptosis, non-programmed death) and functions (E2 synthesis disorder) of GCs are the main cause of follicular atresia [9–11]. Notably, investigations in different female mammals indicated that the states and functions of GCs are controlled by a complicated regulatory network consisting of multiple in vivo and in vitro factors, including micro-environment (hypoxia), toxins (ZEA, BPA), stresses (oxidation), hormones (FSH), growth factors (TGF-β1), homeostasis, cytokines (IL-6), and epigenetic regulators [12, 13]. Among which, non-coding RNAs are an important class of epigenetic regulator, and involved in the regulation of the states and functions of GCs, as well as follicular atresia [14, 15].

Currently, five types of regulatory non-coding RNAs including miRNAs, siRNAs, lncRNAs, circRNAs, and piRNAs have been shown to have the ability to influence follicular development in female mammals [16, 17], among which, the pertaining to miRNAs is more advanced and comprehensive. miRNAs, a class of 19–25 nt endogenous single-strand RNAs, are widely expressed and highly conserved among species [18], which are involved in almost all crucial physiological and pathological processes by regulating the expression of target genes at the DNA and RNA levels in a sequence-complementary pairing manner [19]. Omics-based studies have identified hundreds of miRNAs in follicles, which exhibited strong spatiotemporal specificity throughout follicular development, especially during atresia [20]. In the last decade, multiple functional miRNAs have been identified to regulate GC apoptosis [21], E2 synthesis [22], hormone secretion and response [23, 24], and stress [25]. However, most studies only relied on the classical regulatory mechanism of miRNAs, while not focusing on their basic expression level, the universality of targets and action mode. Therefore, further investigations are needed to identify generalized anti-atretic miRNAs in female mammals.

Through combining multiple high-throughput data, we recently identified an interesting class of miRNAs that is specifically highly expressed in ovary but dramatically downregulated during follicular atresia, indicating that they may maintain the characteristics of ovary tissue and exert anti-atresia effects, which were termed as the downregulated ovary-elevated (OE) miRNAs. After comprehensive analyses of reproductive trait-associated QTL localization, atretic follicle feature correlation, biological function prediction, and expression alteration patterns among species, miR-184, as the most notable downregulated OE miRNA, is selected for further investigation. However, little is known about the biological functions and underlying mechanisms of downregulated OE miRNAs (including miR-184) in ovary. The aim of this study is to explore the anti-atresia properties, functional target genes, and regulatory mode of miR-184, as well as its downregulation mechanism during follicular atresia at the molecular, cellular, and follicular levels. The results will expand the epigenetic mechanisms of follicular atresia, which is contribution to maintaining follicular development and improving ovary health in female mammals.

## Materials and methods

### Animal and ethics

A total of 520 healthy and sexually mature Duroc×Yorkshire×Landrace (DLY) sows (average mass 110 kg and 180 d) were randomly selected from Zhushun Biological Technology Co (Nanjing, China) for bilateral ovaries collection and the isolation and in vitro culture of follicles and GCs in this study (Table S1). Among which, 12 tissue samples (heart, liver, spleen, lung, kidney, pancreas, brain, stomach, intestine, musculature, ovary, and uterus) from three sows were collected and placed in liquid nitrogen for tissue expression profiling analysis. The studied sows were healthy, well taken cared, and slaughtered in accordance with animal welfare regulations. All the animal-involved experiments were reviewed, approved and supervised by the Animal Ethics Committee of Nanjing Agricultural University, Jiangsu, China (NJAU No. 20223024059).

### Follicle isolation, classification, and culture in vitro

Fresh ovaries from DLY sows were soaked in 37 °C PBS and sent back to the laboratory within 1 h. Follicles were isolated and classified as previously described [15]. Specifically, the colorless follicles with more dissociative GCs (>2500/μL), low E2/P4 index, and no vessel on surface were considered as atretic follicles. While, the preantral and antral follicles were recognized via morphological observation (size and cavity formation) and E2 level in follicular fluid. Only the follicles with morphology consistent with steroid hormone levels were selected for following experiments. In vitro culture and treatment of the isolated follicles were performed based on the published study [22].

### Cell culture and treatment

Sow GCs from 3 to 5 mm healthy follicles were isolated using syringes with 22-gauge needles, washed with PBS, and seeded into culture plates filled with DMEM/F12 containing 10% fetal bovine serum (FBS, v/v, #10099, Gibco) and 1% penicillin-streptomycin (PS, v/v, #15140, Gibco). Human GC cell line (KGN) and embryonic kidney cell line (HEK-293T) were purchased from ATCC, and cultured in DMEM with 10% FBS (v/v) and 1% PS (v/v). All cells were confirmed to be mycoplasma negative, and placed in a 37 °C humid incubator with 5% CO_2_. After cultured for 24 h, 3.2 μg plasmids or 50 nM oligonucleotides were transfected into cells using Lipofectamine 3000 reagent (#L13778, Invitrogen). For TGF-β1 and inhibitor treatment, cells were cultured with FBS-free medium overnight, and TGF-β1 (#P01137, Novoprotein), SB431542 (#S1067, Selleck), 5-AzaC (#53160ES50, Yeasen), BCI-121 (#HY-21972, MCE), and ITSA-1 (#HY-100508, MCE) were dissolved with DMSO and added into the medium to the indicated concentration. The oligonucleotides used here were synthesized by GenePharma (Shanghai, China) and are listed in Table S2.

### Bioinformatic analysis

High-throughput data including RNA-seq and microarray were downloaded from NCBI SRA database, and ten cohorts were referred in this study. Sow ovary-elevated (OE) miRNAs were identified based on cohort 1, and the downregulated OE miRNAs during follicular atresia were identified with the criteria as FDR < 0.05 and |Log_2_(fold change)| > 1. The miRNAs located in the QTLs associated with sow reproduction traits were analyzed using Pig QTLdb. Genome location of *miR-184* among different species and their neighbouring genes were analyzed using Ensembl database. The sequences of pre- and mature miR-184 were obtained from miRBase database. DNAMAN and MEGA v5.1 software were applied to evaluate the consistency and evolutionary conservation of pre-miR-184. Tissue expression profiles of hsa-miR-184 were obtained from GTEx and miRGator v3.0 databases. Candidate targets of miR-184 were comprehensively predicted by TargetScan, micro T-CDS, and miRDB. Potential functions and associated signaling pathways of miR-184 were analyzed by DAVID and KOBAS through Gene Ontology (GO) and Kyoto Encyclopedia of Genes and Genomes (KEGG) analyses. miR-184 responsive elements within the promoter of *SMAD3* and SMAD3 mRNA were analyzed using RNAhybrid. The transcription factors (TF) potentially target the promoter of *miR-184* were predicted by JASPAR and ALGGEN database. Characters of *miR-184* promoter, including methylation sites and histone modification, were analyzed by MethPrimer, MethBank, and Ensembl database. Sequence alignment was conducted using ESPript 3.0 and CLUSTALW. The secondary structure of SMAD3 mRNA was analyzed by Mfold. The information of ten cohorts and website address of online database used in this study are listed in Table S3.

### RNA extraction and reverse transcription quantitative PCR (RT-qPCR)

Total RNA from cultured cells was extracted using TRIzol reagent (#R411, Vazyme) and reverse transcribed into cDNA with HiScript III 1st Strand cDNA Synthesis Kit (#R312, Vazyme). Expression of interested genes were quantified using qPCR analysis on Applied Biosystems QuantStudio7 system with a standard protocol from AceQ qPCR SYBR Green PCR Master Mix Kit (#Q111, Vazyme). For the quantitative analysis of miRNAs, 0.5 μg total RNA was used for cDNA synthesis with specific stem-loop primers. The expression levels of protein-coding genes and miRNAs were calculated using the 2^−∆∆CT^ method with the normalization to those of *GAPDH* and *U6*, respectively. All qPCR reactions were repeated three times with three independent samples. The primers used here are listed in Table S4.

### Apoptosis detection

Cell apoptosis was detected by flow cytometry analysis and terminal deoxynucleotidyl transferase dUTP nick end labeling (TUNEL) assay. For flow cytometry analysis, cells after treatment were double stained with Annexin V-FITC and PI (#A211-01, Vazyme) in the dark room for 15 min, and detected by fluorescence-activated cell sorting (FACS) on a cell counting machine (Becton Dickinson). Apoptosis rate was analyzed using Flowjo v10.0 software as previously described [26]. TUNEL Apoptosis Kit (#E-CK-A32, Elabscience) was also utilized to assess cell apoptosis. Briefly, cells were washed, permeabilized, labeled, and stained with FITC. Images of stained cells were obtained from a fluorescence laser confocal microscope (ZEISS). TUNEL-positive cells with green staining signals were counted in five nonoverlapping fields and statistically analyzed as percentages.

### Proliferation analysis

Cell proliferation was analyzed using Cell Counting Kit-8 (CCK-8) and 5-ethynyl-2’-deoxyuridine (EdU) assay. For CCK-8 assay, cells were seeded into 96-well plates at a density of 2000 cells per well. After treatment, 10 μL CCK-8 solution (#A311-01, Vazyme) was added to culture medium and incubated at 37 °C for 2 h. The absorbance of each well was measured using a microplate reader with an optical density of 450 nm. EdU assay was performed using the BeyoClick™ EdU-488 Cell Proliferation Detection Kit (#K1076, APExBIO). Briefly, cells were seeded on coverslips at a density of 10,000 cells per coverslip. After washing, fixation, permeabilizing, EdU labeling, and nuclear staining, the red fluorescence was observed under a fluorescence laser confocal microscope (ZEISS) at 488 nm wavelengths. The percentage of EdU-positive cells in five different microscopic fields was statistically calculated.

### Morphometric analysis

After treatment for 48 h, cell images were obtained from a Leica inverted microscope to evaluate the effects of miR-184 on the morphology of sow GCs. Specifically, cells with clear edges with no visible serrations or breaks were considered to have high membrane integrity. Conversely, cells with poorly defined smooth edges, obvious internal vesicles, cell shrinkage, or even rupture were considered to be impaired. For each sample, five different microscopic fields were selected and the percentage of impaired cells was statistically analyzed.

### Immunoblotting

Cells were washed with ice-cold PBS and lysed with RIPA lysis buffer containing 10 mM PMSF (#P0013, Beyotime) and protease inhibitors. After centrifuged at 13,000 × *g* for 20 min, protein was collected and the concentration was detected using BCA Kit (#P0012, Beyotime). Immunoblotting assays were performed as previously described [27]. In short, protein were resolved in SDS-PAGE (#M00656, Genscript) and transferred onto PVDF membranes, which were blocked with 5% skim milk and incubated with indicated antibodies at 4 °C for 12 h. After washed with TBST three times, the membranes were incubated with HRP-conjugated secondary antibodies for 2 h at room temperature. Immunodetection was achieved with ECL luminescent solution (#E423, Vazyme) and high-resolution blotting images were obtained from a chemiluminescent imaging system (Bio-Rad). Greyscale quantification was performed using Image J software (NIH, USA), and the protein level of GAPDH was utilized as an internal control. The primary antibodies used here were anti-SMAD3 (#D155234, Sangon Biotech, 1:2000), anti-p-SMAD3 (#D155153, Sangon Biotech, 1:1000), anti-TGF-β1 (#bs-0086R, Bioss, 1:1000), anti-TGFBR2 (#sc-400, Santa Cruz, 1:2000), anti-SMAD4 (#sc-1909R, Santa Cruz, 1:1000), anti-SMAD7 (#D160746, Sangon Biotech, 1:1000), anti-AGO2 (#A6023, ABclonal, 1:1000), anti-SREBF2 (#28212-1-AP, Proteintech, 1:1000) and anti-GAPDH (#10494-1-AP, Proteintech, 1:5000). Each group had three independent biological replicates. Full and uncropped western blots and gel images are detailed in the supplementary material.

### Chromatin immunoprecipitation (ChIP)

ChIP assays were performed as described previously [28]. Briefly, 1 × 10^7^ GCs were fixed with 1% formaldehyde for 10 min at room temperature and quenched with glycine for 10 min. Then, the cross-linked protein-DNA complexes were immunoprecipitated with indicated antibodies. After ultrasonic fragmentation and purification, enrichment of protein-interacting DNA fragments was detected by PCR and qPCR. The antibodies used here were anti-H3K4me2 (#9725, Cell Signaling Technology), anti-H3K9Ac (#9649, Cell Signaling Technology), anti-H3K4me3 (#91264, Proteintech), and anti-AGO2 (#A6023, ABclonal). Anti-IgG antibody (#sc-2358, Santa Cruz, USA) was used as an internal control, and untreated genomic DNA was used as input control. Primers used for ChIP are listed in Table S4.

### Rapid amplification of cDNA end (RACE)

Sequence of *SMAD3* 5′-UTR was identified using RACE with SMARTer RACE 5′/3′ Kit (#634858, Clontech Laboratories Inc.). The gene-specific primer designed for 5′-RACE was shown as follows: 5′-GSP, 5′-TGG TTC ATC TGG TGG TCG CTG GTC T-3′. The corresponding PCR product was analyzed by electrophoresis on a 2.0% agarose gel, and the clear band was collected, purified, and cloned into pClone007 vector (#TSV-007BS, Tsingke) for Sanger sequencing. Finally, the 5′-teminal of *SMAD3* was verified through mapping to *Sus scrofa* RefSeq 11.1.

### RNA pull-down

The biotinylated single-strand RNA transcripts of miR-184 and the 5′-UTR of SMAD3 mRNA with wild-type and mutant miR-184 responsive element were designed and synthesized by Generay Biotechnology Co. (Shanghai, China). RNA pull-down was performed as follows, 10 μg biotin-labeled transcripts were incubated with 30 μg total RNA from sow GCs or KGN cells for 4 h at room temperature, and the biotin-RNA/RNA complexes were pulled down with streptavidin magnetic beads (#D112005, Sangon). After isolation, the interacted RNAs were detected and quantified by RT-qPCR.

### RNA immunoprecipitation (RIP)

GCs were cross-linked with 1% formaldehyde and incubated with RIP buffer at 4 °C for 30 min. The supernatant was precleared after centrifuged at 13,000 × *g* for 20 min, and incubated with 4 μg anti-AGO2 antibody and 100 μL Protein A dynabeads (#10008D, Invitrogen) at 4 °C overnight with head-to-head rotation. Beads were washed with RIP buffer three times 4 °C for 5 min and decrosslinked at 75 °C for 45 min. Finally, the RNAs interacted with AGO2 were extracted using TRIzol reagent, and further identified and quantified by RT-qPCR.

### Histology analysis

Fresh pig ovaries were fixed in PBS with 10% formalin. After dehydrated through an ethanol series, cleared in xylene, and embedded in paraffin according to a standard protocol, the embedded ovarian was cut into 4-μm-thick slices, stained with hematoxylin and eosin, and analyzed by microscopic detection.

### RNA fluorescence in situ hybridization (RNA FISH)

RNA FISH was performed by Servicebio Co. (Wuhan, China). For the quantitative analysis of miR-184 during follicular development, the sliced pig ovary samples were post-fixed, paraformaldehyde, acetylated, and hybridized at 65 °C with the following DIG-labeled anti-sense probe: 5′-ACC CTT ATC AGT TCT CCG TCC A-3′. To detect the subcellular location of miR-184 and its co-localization with SMAD3 mRNA in sow GCs, cells were washed, fixed, permeabilized, and hybridized with the above probe for miR-184 (green) and the following DIG-labeled SMAD3 mRNA probe (red): 5′-AAA CTC CTG GTT GTT GAA GAT CTT CAG GTT-3′. Nuclei was stained with 4,6-diamidino-2-phenylindole dye (DAPI). Images were obtained from fluorescence microscopy using a Nikon Eclipse 80i microscope equipped with a Nikon DS-2 digital camera.

### Plasmids construction

To determine the core promoter of *ssc-miR-184*, different fragments (−1925/−18, −1574/−18, −1159/−18, −670/−18, and −240/−18) were amplified, purified and inserted into the pGL3-Basic vector (#E1751, Promega) between *Nhe*I and *Xho*I restriction enzyme sites. To analyze the effects of TFs on the transcription activity of *miR-184*, its core promoter with the wild-type binding motifs was synthesized by TsingKe (Nanjing, China), and inserted into pGL3-Basic vector. To detect the influence of miR-184 on the transcription activity of *SMAD3*, its promoter containing miR-184 responsive element was synthesized and inserted into the pGL3-Basic vector. To access whether miR-184 directly targets SMAD3 mRNA, the 5′-UTR and 3′-UTR of SMAD3 mRNA with miR-184 binding sites were synthesized and inserted into the pGL3-promoter (#E1761, Promega) and pmirGLO (#E1330, Promega) between *Sac*I and *Xba*I restriction sites, respectively. For mutation, homologous recombination was conducted using Trelief® SoSoo Cloning Kit (#TSV-S2, TsingKe) with the vectors mentioned above as substrates. All the recombinant vectors were verified by Sanger sequencing.

### Luciferase reporter assay

Cells were collected after transfection for 24 h, and the luciferase activities (*Firefly* and *Renilla*) of each sample were measured by GLOMAX detection system (Promega) using a Dual-Luciferase Reporter Kit (#E1910, Promega). Relative luciferase activity of each sample was calculated as the ratio of *Firefly*/*Renilla*.

### E2, P4, and Caspae3 activity detection

The concentration of estradiol (E2) and progesterone (P4) in the follicular fluid (FF) were measured using an Estradiol Detection Kit (#ARE-8800, BNIBT) and a Progesterone Detection Kit (#FRE-2500) with ELISA method, respectively. Briefly, the FF samples were collected and 10-fold diluted. After reaction and termination, the optical density of each sample was detected under 450 nm wavelength, which was converted to the concentration of E2 and P4. The activities of Caspase3 were measured using a Caspase3 Activity Detection Kit (#C1116, Beyotime). In short, sow GCs under different conditions were collected and lysed. After centrifuged for 10 min, the supernatant was incubated with 2 mM Ac-DEVD-ρNA at 37 °C for 1.5 h, the optical density was detected under 405 nm wavelength, which was converted to the Caspase3 activity based on the standard curve.

### RNA polymerase II inhibitor ActD chase assay

For SMAD3 mRNA stability analysis, sow GCs and KGN cells were transiently transfected with miR-184 mimics for 24 h. Then, Actinomycin D (ActD) was added to the culture medium to a final concentration of 10 mg/mL and incubated for 2 h, 4 h, and 6 h, respectively. After incubation, cells were collected and the stability of SMAD3 mRNA was detected using qPCR. To detect the stability of luciferase (LUC+) mRNA, pGL3-SMAD3-5′UTR was transfected with miR-184 mimics into HEK-293T cells for 24 h. LUC+ mRNA stability was analyzed by qPCR after incubation with 10 mg/mL ActD for the indicated times.

### Subcellular fractionation

To detect the subcellular location of miR-184, GCs were lysed with cold lysis buffer (PBS with 0.1% NP-40 and 10 mmol/L RNAase inhibitor) for 10 min. After centrifuged at 5000 × *g* for 3 min at 4 °C, the supernatant was aspirated to extract cytoplasmic RNA. Precipitated solids were resuspended by adding 500 µL lysis buffer for 10 min, and the supernatant was discarded after centrifuged for 3 min at 4 °C. The precipitated solid was considered as the nucleus fraction and collected for nuclear RNA extraction. Subcellular localization of miR-184 was analyzed by qRT-PCR with above-montioned RNAs as substrates, in which *U6* and *GAPDH* subcellular distribution levels were validated for the nucleus and cytoplasm, respectively.

### Statistical analysis

Experiments in this study were conducted in triplicates with at least three independent samples. Statistical analyses, including significance calculation and Pearson correlation were conducted and visualized using GraphPad Prism v8.0, IBM SPSS Statistics v26.0, and RStudio v4.1. Data were shown as mean ± S.E.M. Significance between two or multiple groups were analyzed using two-tailed independent Student’s *t*-test and one-way analysis of variance (ANOVA). The stability and accuracy of the risk model were estimated by receiver operating characteristic curve (ROC) and corresponding area under the curve (AUC) value. *P < 0.05 were considered to be statistically significant.

## Results

### Identification of the downregulated OE miRNAs during follicular atresia

**Fig. 1:**
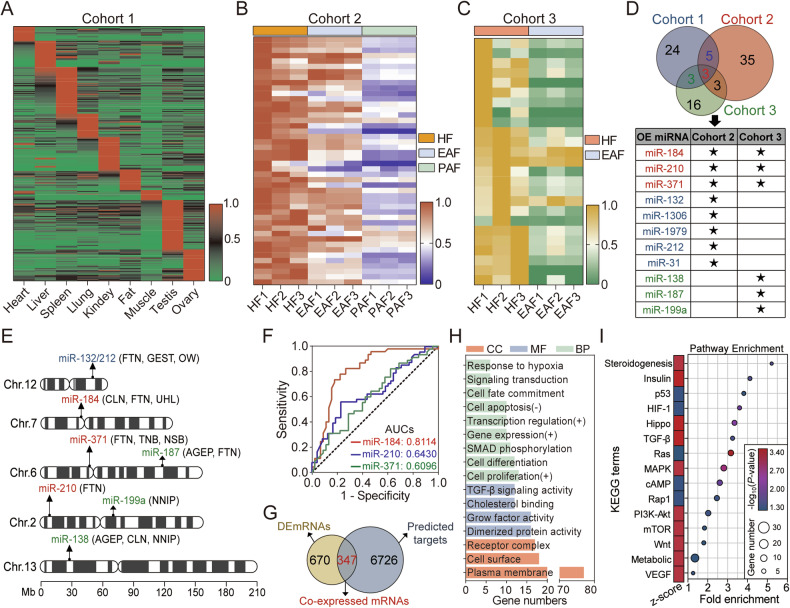
Identification and characterization of the downregulated OE miRNAs. **A** Heatmap showing the tissue expression profile of 557 function-known miRNAs in pig from Cohort 1. Red and green indicate high and low expression, respectively. **B**, **C** Heatmap depicting the expression pattern of 46 and 25 downregulated miRNAs in sow ovarian follicles during atresia from Cohort 2 (**B**) and Cohort 3 (**C**). HF, EAF, and PAF indicate healthy, early atretic, and progressed atretic follicle, respectively. **D** Venn diagram showing the downregulated OE miRNAs during follicular atresia. Black stars indicate the miRNAs identified in corresponding cohort. **E** The chromosome pattern diagram showing the downregulated OE miRNAs located within pig reproduction trait-associated QTLs. **F** ROC curves and AUC values of miR-184, miR-210, and miR-371 expression levels associated with sow follicular atresia (*n* = 45). **G** Identification of the downregulated OE miRNA-involved co-expressed mRNAs. **H**, **I** Potential functions and enriched signaling pathways of the downregulated OE miRNAs were analyzed by GO (**H**) and KEGG (**I**) analyses. The columns in red, blue, and green indicate CC (cell component), MF (molecular function), and BP (biological process) categories. Z-scores were calculated to estimate the alteration trend of the enriched KEGG terms, which were shown as rectangles labeled in red (activation) and blue (inactivation).

In order to identify the downregulated ovary-elevated (OE) miRNAs during follicular atresia, OE miRNAs were firstly characterized using a miRNA expression profile with nine pig tissues (Cohort 1). Tissue expression pattern of 557 function-known miRNAs were analyzed and 35 of which specifically highly expressed in ovary were considered as OE miRNAs (Fig. 1A, Table S5). Next, the significantly downregulated miRNAs in sow ovarian follicles during atresia were identified using our previous microarray (Cohort 2) and RNA-seq (Cohort 3) data [20, 29]. As shown in Fig. 1B, C, 46 and 25 miRNAs were respectively identified based on the following properties: |Log_2_(fold change)| ≥ 1, FDR < 0.05, and signal >100 or TPM > 10. After comprehensive analysis, 11 miRNAs were considered as downregulated OE miRNAs during follicular atresia, and among which 3 miRNAs (miR-184, miR-210, and miR-371) were notably detected in both cohorts (Fig. 1D). Global analysis of the miRNAs associated with QTLs was conducted and showed that 250 miRNAs including the above-mentioned OE miRNAs (except for miR-1306) located in pig reproduction trait-associated QTLs, such as litter size (LS), corpus luteum number (CLN), uterine horn length (UHL), and number of stillborn (NSB), indicating that OE miRNAs potentially regulate the development of follicle, uterus, and fetal (Fig. 1E, Fig. S1, and Table S6). Besides, a miRNA-dependent ROC curve was established to access the expression levels of three common downregulated OE miRNAs in GCs to predict follicular atresia (Fig. 1F). Results showed that AUC of miR-184 was 0.811, revealing high prediction accuracy of sow follicular atresia by referring to the expression of miR-184. In addition, the functions of downregulated OE miRNAs were analyzed using GO and KEGG analyses based on their co-expressed protein-coding mRNAs (Fig. 1G). A total of 84 GO terms and 17 KEGG pathways related to cell states, stress response, gene expression, signal transduction, and steroidogenesis were significantly enriched (Fig. 1H, I and Tables S7, S8). These findings indicate that downregulated OE miRNAs may be involved in the initiation and progression of sow follicular atresia.

### miR-184, a highly conserved OE miRNA, shows low expression in atretic follicles

**Fig. 2:**
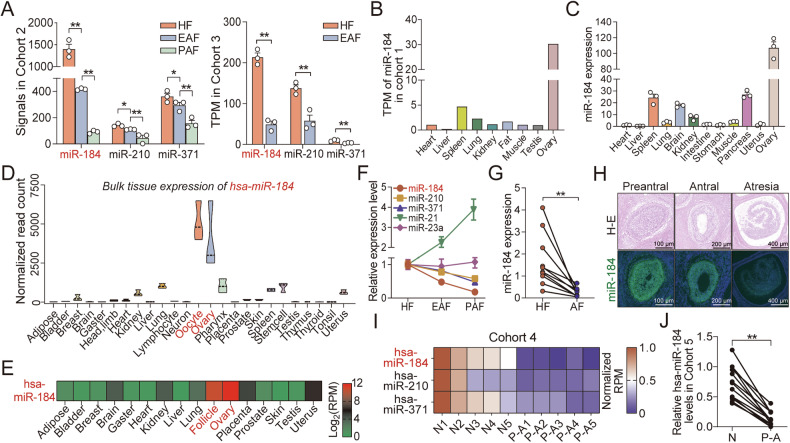
miR-184, a highly conserved OE miRNA, is dramatically downregulated in atretic follicles. **A** The expression of miR-184, miR-210, and miR-371 in sow follicles at different stages from Cohort 2 (left panel) and Cohort 3 (right panel) were analyzed (n = 3). **B** miR-184 expression in nine pig tissues from Cohort 1. **C** miR-184 expression in 12 pig tissues were detected by RT-qPCR (n = 3). **D**, **E** Human tissue expression profile of hsa-miR-184 from GTEx (**D**) and DIANA-miTED database (**E**). **F** The expression of miRNAs in sow follicles during atresia was analyzed by RT-qPCR (n = 3). **G** miR-184 levels in ten pairs of sow healthy and atretic follicles were examined by RT-qPCR. **H** miR-184 expression in sow follicles at different stages was detected by HE staining and RNA FISH. **I** Heatmap showing the normalized RPM of hsa-miR-184 in human normal follicles (N, n = 5) and PCOS-mediated atretic follicles (P-A, n = 5) from Cohort 4. **J** Hsa-miR-184 levels in 13 pairs of human normal and PCOS-mediated atretic follicles from Cohort 5. Data were shown as mean ± SEM with at least three independent replicates. Significance in (**A**) were analyzed by unpaired two-tailed Student’s *t*-test, in (**G**) and (**H**) by paired two-tailed Student’s *t*-test. *P < 0.05, **P < 0.01.

Until now, the roles of downregulated OE miRNAs in the regulation of follicular atresia remain elusive. Based on the ROC analysis, functional prediction, and expression alteration pattern during follicular atresia (Fig. 2A), miR-184 was selected for the following investigation. Firstly, the sequences of pre- and mature miR-184 from 11 species were aligned and showed high consistency (87.35% and 99.75%) (Fig. S2A). Chromosome location analysis found that the neighboring protein-coding genes of *miR-184* from different species were completely identical (*RASGRF1* and *ANKRD34*) (Fig. S2B). Besides, through MEGA analysis, miR-184 among species had high evolutionary conservation (Fig. S2C), indicating that miR-184 is a highly conserved miRNA. Previous omics study showed that miR-184 exhibited the highest expression in ovary tissue (Fig. 2B), which was verified by RT-qPCR in this study with 12 sow tissues (Fig. 2C). Notably, similar phenomenon was also existed in women, and we found that hsa-miR-184 is highly expressed in ovary tissue, including follicle and oocyte, through GTEx and DIANA-miTED database (Fig. 2D, E). To verify the expression pattern of miR-184 during follicular atresia, RT-qPCR and FISH were performed and showed that it was dramatically downregulated in sow atretic follicles (Fig. 2F–H). Interestingly, low expression of miR-184 was also identified in women PCOS-mediated atretic follicles and bovine large atretic follicles from published omics data (Cohort 4-7) (Fig. 2I, J and Fig. S3). These results indicate that low expression of miR-184 may be an inducer of the follicular atresia in mammals.

### Downregulated miR-184 induces apoptosis and inhibits proliferation of follicular GCs

**Fig. 3:**
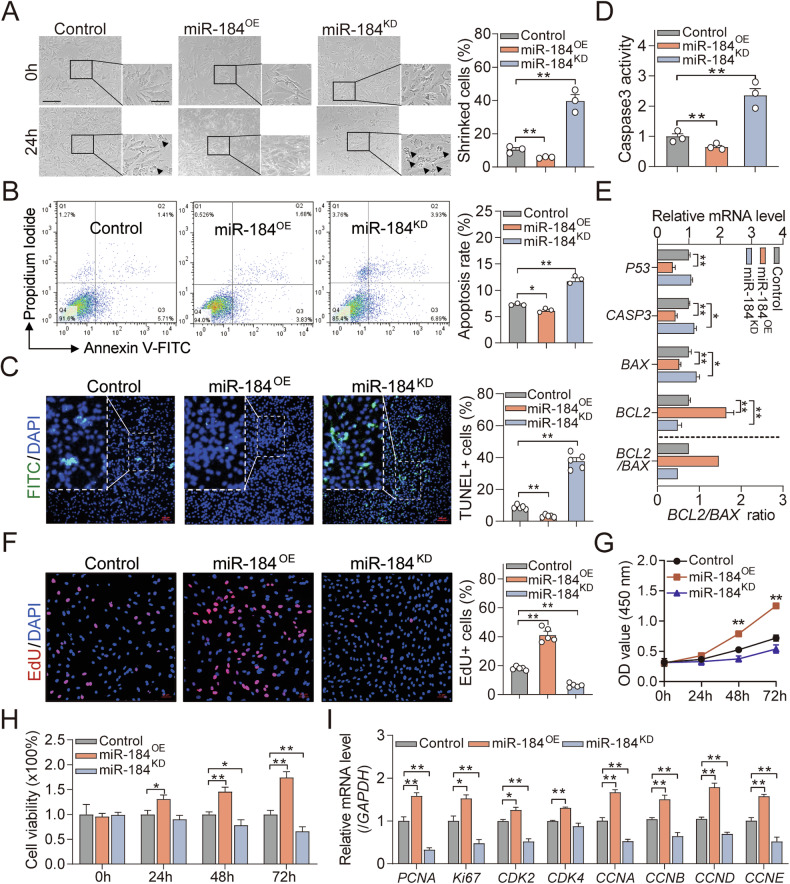
miR-184 inhibits the apoptosis and induces the proliferation of sow GCs. **A**–**I** miR-184 was over-expressed (OE) or knocked down (KD) in sow GCs after transfection with miR-184 mimics or specific inhibitor, and cell morphology (shrinked cells were indicated by black arrows) was analyzed by morphometric observation (**A**), cell apoptosis was detected by FACS (**B**) and TUNEL (**C**), Caspase3 activity was measured by ELISA (**D**), the expression of apoptosis-related genes and *BCL2*/*BAX* ratio were quantified by RT-qPCR (**E**), cell proliferation and viability were analyzed by EdU staining (**F**) and CCK-8 (**G**, **H**), and the expression of cell cycle and proliferation-related genes were detected by RT-qPCR (**I**). Data were shown as mean ± SEM with at least three independent replicates. Significance in (**A**–**I**) were analyzed using ANOVA with post-*hoc* multiple comparisons. *P < 0.05, **P < 0.01.

The above RNA FISH assay showed that miR-184 was mainly located in GCs (Fig. 2H), which is essential for the fate of follicles in female mammals [30], suggesting that miR-184 may affect follicular development by regulating GC state. To further investigate the anti-atretic effect of miR-184 in follicles, miR-184 mimics or specific inhibitors were transfected into GCs cultured in vitro, respectively (Fig. S4A). Through multi-dimension analyses including morphometric observation, FACS, TUNEL, ELISA, and RT-qPCR, we found that knockdown of miR-184 impaired cell morphology and membrane integrity (Fig. 3A), induced GC apoptosis (Fig. 3B, C), elevated Caspase3 activity (Fig. 3D), increased *P53* and *Caspase3* expression, and reduced *BCL2*/*BAX* ratio (Fig. 3E), while the opposite results were occurred in miR-184 over-expressed GCs, indicating that miR-184 acts as an anti-apoptotic miRNA in sow GCs. Besides, through EdU, CCK-8, and RT-qPCR assays, we also noticed that inhibition of miR-184 impeded cell proliferation (Fig. 3F, G), suppressed cell viability (Fig. 3H), and influenced the expression of cell proliferation and cycle related genes, such as *PCNA*, *Ki67*, *CDK2, CCNB, CCND*, and *CCNE* (Fig. 3I). However, miR-184 over-expressed GCs showed the opposite phenotype. Similarly, the anti-apoptotic and proliferative effects of miR-184 were also noticed in KGN cells, a well-known human ovarian GC line (Fig. S4B–D). The above findings strongly indicate that downregulation of miR-184 induces apoptosis and inhibits proliferation of GCs in sows and women.

### SMAD3 is a positive functional mediator of miR-184 in sow GCs

**Fig. 4:**
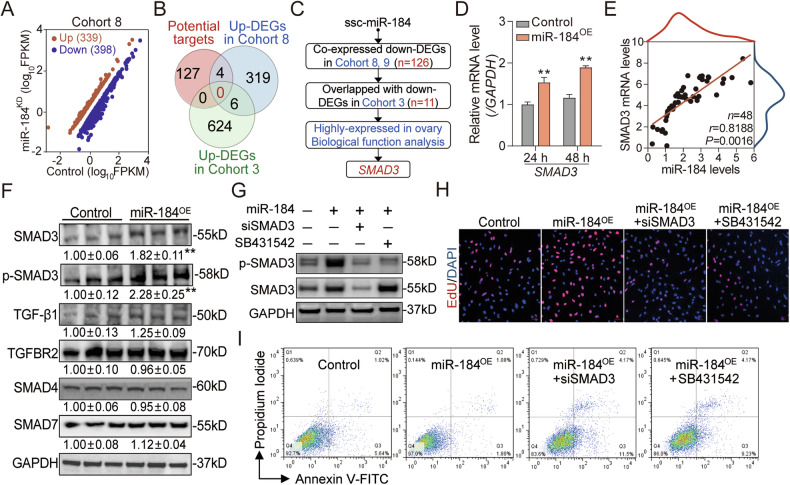
miR-184 exerts anti-apoptotic functions by inducing *SMAD3* expression and activating TGF-β signaling pathway. **A** Scatter plot showing the DEGs in sow GCs after miR-184 inhibition by RNA-seq. **B** No potential functional target of miR-184 was predicted based on bioinformatics analysis and the upregulated DEGs in Cohort 3 and Cohort 8. **C** Strategy for identification of the candidate functional mediators of miR-184 via co-expressed gene-function network construction, expression and function feature analysis. **D** Effects of miR-184 mimics on SMAD3 mRNA levels in sow GCs were analyzed using RT-qPCR (*n* = 3). **E** The expression correlation between miR-184 and SMAD3 in sow follicles was detected using Pearson correlation analysis (*n* = 48). **F** miR-184 mimics were transfected into GCs for 48 h, and the protein levels of SMAD3, p-SMAD3, TGF-β1, TGFBR2, SMAD4, and SMAD7 were measured using western blotting (*n* = 3). **G**–**I** miR-184 mimics were co-transfected into GCs with SMAD3-siRNA or SB431542 for 48 h, then SMAD3 and p-SMAD3 protein levels were assessed by western blotting (**F**), cell proliferation was analyzed by EdU (**G**), and cell apoptosis was detected by FACS (**H**). Data were shown as mean ± SEM with at least three independent replicates. Significance were analyzed using two-tailed Student’s *t*-test. **P < 0.01.

To elucidate the functional mechanisms of miR-184 in sow GCs, RNA-seq (Cohort 8) was performed and 737 differentially expressed genes (DEGs) were identified in GCs after miR-184 inhibition (Fig. 4A). However, no functional target potentially mediates the anti-atretic effect of miR-184 was recognized in combination with bioinformatics analysis and omics data (Fig. 4B). We next established the miR-184-mediated co-expressed gene-function network and found that *SMAD3*, a key regulatory gene for follicular development which is highly expressed in GCs, is a candidate functional mediator of miR-184 (Fig. 4C). Quantitative analysis showed that *SMAD3* expression in sow GCs was significantly elevated by miR-184 mimics (Fig. 4D). Pearson correlation analysis also revealed a significantly positive correlation between the expression of *miR-184* and *SMAD3* in follicles (Fig. 4E). Thus, *SMAD3* was selected for the following investigation. Western blotting assay showed that overexpression of miR-184 significantly increased SMAD3 protein level and TGF-β signaling pathway activity (p-SMAD3 levels), while had no effect on the ligand (TGF-β1), receptor (TGFBR2), I-SMAD (SMAD7), and co-SMAD (SMAD4) members of the canonical TGF-β signaling pathway (Fig. 4F), indicating that miR-184 specifically induces the expression and activity of SMAD3. To further illustrate whether SMAD3 or TGF-β signaling pathway mediate the function of miR-184, SMAD3-siRNA and SB431542 (TGF-β signaling pathway inhibitor) were applied in FACS and CCK-8 assays. As shown in Fig. 4G–I, knockdown of SMAD3 or inactivation of TGF-β signaling pathway notably disrupted the proliferative and anti-apoptotic function of miR-184 in sow GCs. Moreover, our previous RNA-seq data (Cohort 10) and RT-qPCR result showed that miR-184 was not regulated by TGF-β1 (Fig. S5A, B), but inhibition of miR-184 notably impaired TGF-β1-meidated pathway activity and biological functions in sow GCs (Fig. S5C–E). Taken together, these results demonstrate that SMAD3 is a specific and positive functional mediator of miR-184, while miR-184 maintains the normal state of GCs through inducing *SMAD3* expression and elevating TGF-β signaling pathway activity.

### miR-184 induces *SMAD3* expression at both transcriptional and post-transcriptional level

**Fig. 5:**
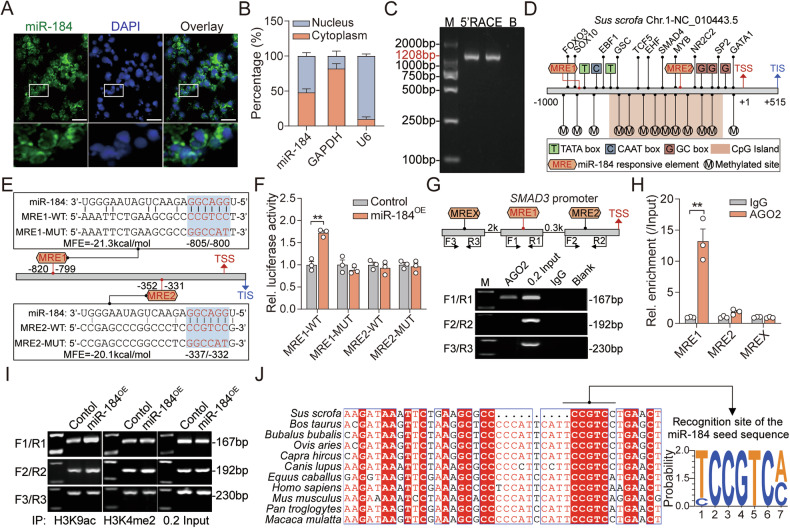
miR-184 induces *SMAD3* transcription in GCs by acting as a saRNA. **A**, **B** The subcellular localization of miR-184 in sow GCs was analyzed by RNA FISH (**A**) and subcellular fractionation (**B**). Scale: 20 μm. **C** Gel image showing the 5′-RACE amplicon of pig *SMAD3*. **D** Diagram depicting the potential *cis*-acting elements, methylation sites, TF motifs, and miR-184 responsive elements within the promoter of *SMAD3*. **E** Diagram showing the recombination vectors containing *SMAD3* promoter with wild-type (WT) or mutant (MUT) MREs. **F** Effect of miR-184 on the transcriptional activity of *SMAD3* was analyzed using dual luciferase activity assay. **G**, **H** Enrichment of AGO2 on *SMAD3* promoter was measured using ChIP (**G**) and ChIP-qPCR (**H**). **I** H3K4me2 and H3K9ac levels on the promoter of *SMAD3* in miR-184 over-expressed GCs were detected using ChIP. **J** Sequence alignment of miR-184 responsive element within *SMAD3* promoter among different mammals. Data were shown as mean ± SEM with at least three independent replicates. Significance were analyzed using two-tailed Student’s *t*-test. **P < 0.01.

To investigate the regulatory mechanisms of miR-184 to *SMAD3* expression in sow GCs, we analyzed the subcellular localization of miR-184 through FISH and nucleoplasmic separation, and found that it evenly distributed in both nucleus and cytoplasm (Fig. 5A, B), suggesting that miR-184 in the nucleus induces *SMAD3* transcription through acting as a small activating RNA (saRNA). To address this, RACE was performed to detect the transcription start site (TSS) and candidate promoter of *SMAD3* (Fig. 5C). Characterization analyses showed that several *cis*-acting elements (TATA and CAAT box), methylation sites, TF binding motifs, and two potential miR-184 responsive elements (MRE1 and MRE2) located within *SMAD3* promoter (Fig. 5D). Next, the recombination vectors containing *SMAD3* promoter with wild-type (WT) or mutant (MUT) MREs were constructed, and the results of luciferase activity assay indicated that miR-184 overexpression significantly elevated the activity of MRE1-WT vector, while had no effect on the activity of MRE1-MUT, MRE2-WT, and MRE2-MUT vectors (Fig. 5E, F), indicating that miR-184 induces *SMAD3* transcription via MRE1. Furthermore, ChIP and ChIP-qPCR were performed and showed that AGO2, a core member of miRNA-induced regulatory complex, specifically interacted with MRE1, rather than MRE2 (Fig. 5G, H). Besides, we also noticed that miR-184 could significantly induce the histone modification on *SMAD3* promoter, including H3K4me2 and H3K9Ac (Fig. 5I). In addition, alignment analysis showed that the motifs of MRE1 on the promoter of *SMAD3* from different vertebrates are highly conserved (Fig. 5J), suggesting that it is a universal mechanism for the regulation of miR-184 to *SMAD3*, which was further verified in KGN cells through multi-dimension assays (Fig. S6A–D). Altogether, these findings demonstrate that miR-184 in the nucleus specifically binds to *SMAD3* promoter, further alters histone modification and induces *SMAD3* transcription in GCs through saRNA mechanism.

**Fig. 6:**
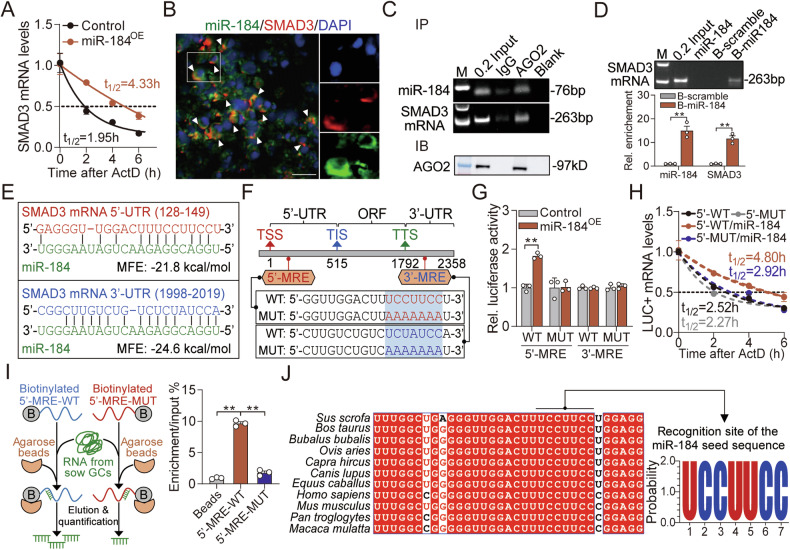
miR-184 stabilizes SMAD3 mRNA by directly binding to its 5′-UTR. **A** The effect of miR-184 on the stability of SMAD3 mRNA was detected by ActD chase assay. **B** Confocal micrographs showing the co-localization of miR-184 (green) and SMAD3 mRNA (red) in GCs. Scale: 10 μm. **C** Enrichment of SMAD3 mRNA on AGO2 was measured using RIP assay. **D** Interaction between miR-184 and SMAD3 mRNA in GCs was verified by in vitro RNA pull-down (*n* = 3). B indicates biotin. **E** RNA hybridization was analyzed to predict the potential MRE of miR-184 on SMAD3 mRNA. **F** Recombination vectors construction. **G** The effects of miR-184 on the 5′-UTR and 3′-UTR activity of SMAD3 mRNA were analyzed by dual luciferase activity detection (*n* = 3). **H** The stability of LUC+ mRNA in miR-184 over-expressed GCs was detected by ActD chase assay. **I** The responsive element of miR-184 within the 5′-UTR of SMAD3 mRNA was verified by in vitro RNA pull-down. B indicates biotin. **J** Sequence alignment of miR-184 responsive element within the 5′-UTR of SMAD3 mRNA among mammals. Data were shown as mean ± SEM with at least three independent replicates. Significance were analyzed using two-tailed Student’s *t*-test or ANOVA. **P < 0.01.

Recently, miRNAs in the cytoplasm are reported to play an important role in maintaining the stability of target mRNAs [31]. To further evaluate the effect of miR-184 on the stability of SMAD3 mRNA, RNA polymerase II inhibitor ActD chase assay was performed and showed higher stability of SMAD3 mRNA in miR-184 over-expressed GCs (Fig. 6A), indicating that miR-184 stabilizes SMAD3 mRNA. Besides, RNA FISH showed the co-localization of miR-184 and SMAD3 mRNA in the cytoplasm of GCs (Fig. 6B), and RIP results revealed that AGO2 bind to SMAD3 mRNA (Fig. 6C). In addition, in vitro RNA pull-down demonstrated that miR-184 directly bind to SMAD3 mRNA (Fig. 6D). To verify the binding sites, RNA hybrid analysis was applied and two potential MREs were predicted within the 5′-UTR and 3′-UTR of SMAD3 mRNA, rather than CDS region (Fig. 6E). Next, luciferase activity detection combined with ActD chase assay indicated that miR-184 could only induce the 5′-UTR activity and stability of SMAD3 mRNA with wild-type 5′-MRE, while had no effect on the vector with 3′-MRE or mutant 5′-MRE (Fig. 6F–H). As expected, in vitro RNA pull-down validated that the 5′-MRE is indispensable for the physical interaction between SMAD3 mRNA and miR-184 (Fig. 6I). Interestingly, alignment analysis showed that the MRE within the 5′-UTR of SMAD3 mRNA are highly conserved among mammals (Fig. 6J). Combined with the analyses in KGN cells demonstrate that it is a universal mechanism for the regulation of miR-184 to SMAD3 mRNA (Fig. S6E–G). Collectively, our findings reveal that miR-184 in the cytoplasm maintains the stability of SMAD3 mRNA by interacting with its 5′-UTR.

### *miR-184* is transcriptionally activated by SREBF2 in an H3K4me3-dependent manner

**Fig. 7:**
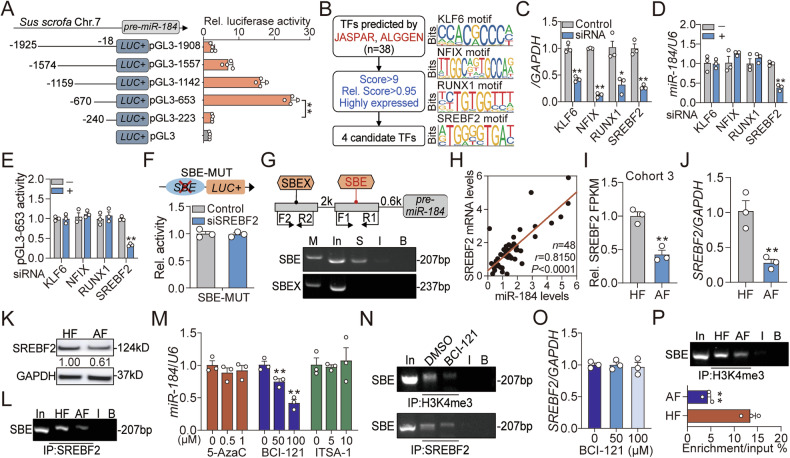
*miR-184* is transcriptionally activated by SREBF2 in an H3K4me3-dependent manner. **A** The core promoter of *miR-184* was identified by truncated promoter analysis and luciferase activity assay (*n* = 3). **B** Flow chart showing the strategy for prediction of the TFs that potentially target the core promoter of *miR-184*, and their motifs were indicated. **C** Knockdown efficiency detection (*n* = 3). **D**, **E** Expression level (**D**) and promoter activity (**E**) of *miR-184* in GCs after knockdown of each TF for 48 h were analyzed using qPCR and luciferase activity assay (*n* = 3). **F** Effect of *SREBF2* knockdown on the promoter activity of *miR-184* with mutant SBE was evaluated by luciferase activity assay (*n* = 3). **G** SREBF2 enrichment on the core promoter of *miR-184* was measured using ChIP. In, I, and B indicate input, IgG, and blank, respectively. **H** Expression correlation between SREBF2 mRNA and miR-184 in follicles was identified by Pearson correlation analysis (*n* = 48). **I**–**K** SREBF2 expression levels in HF and AF were comparatively analyzed using RNA-seq (**I**), RT-qPCR (**J**) and western blotting (**K**) (*n* = 3). **L** ChIP was employed to quantify the enrichment of SREBF2 on the core promoter of *miR-184* in GCs from HF and AF. **M** miR-184 levels in GCs treated with 5-AzaC (0, 0.5, 1 μM), BCI-121 (0, 50, 100 μM) or ITSA-1 (0, 5, 10 μM) for 48 h were detected using qPCR (*n* = 3). **N** Effects of BCI-121 on the enrichment of H3K4me3 and SREBF2 on the core promoter of *miR-184* were analyzed using ChIP. **O** The mRNA levels of SREBF2 in GCs treated with BCI-121 (0, 50, 100 μM) for 48 h were quantified using qPCR (*n* = 3). **P** H3K4me3 enrichment on the core promoter of *miR-184* in GCs from HF and AF were analyzed by ChIP and ChIP-qPCR (*n* = 3). Data were shown as mean ± SEM. Significance were analyzed using two-tailed student’s *t*-test or ANOVA. *P < 0.05, **P < 0.01.

To explore the downregulation mechanism of miR-184 in GCs during follicular atresia, its transcriptional mode (dependent or independent on host gene) was first analyzed. Through basic expression level detection, alteration pattern identification, and siRNA-based qPCR analysis showed that *miR-184* was transcribed independently (Fig. S7A–D). Next, detailed truncated promoter analysis was performed and −670/−240 nt region was identified as the core promoter of *miR-184* (Fig. 7A). After JASPAR and ALGGEN prediction, four candidate TFs with high scores were found potentially bind to the core promoter of *miR-184*, including KLF6, NFIX, RUNX1, and SREBF2 (Fig. 7B). To determine their regulatory roles on *miR-184* transcription, these putative TFs were respectively knocked down in GCs (Fig. 7C). It was noteworthy that the expression and transcription activity of *miR-184* were dramatically inhibited after SREBF2 knockdown, rather than other TFs (Fig. 7D, E). To verify the SREBF2 binding element (SBE), mutation (SBE-MUT) was introduced and showed that its activity was not influenced by SREBF2-siRNA (Fig. 7F), indicating that SBE (−612/−603) is responsible for SREBF2 recognition. As expected, ChIP assay confirmed that SREBF2 directly bind to the SBE on the core promoter of *miR-184* (Fig. 7G). Interestingly, alignment analysis showed that the motifs of SREBF2 within *miR-184* promoter among mammals are highly conserved (Fig. S8). Pearson correlation analysis revealed a positive expression correlation between SREBF2 and miR-184 in GCs (Fig. 7H). Taken together, these findings demonstrate that SREBF2 induces *miR-184* transcription in GCs by binding to its core promoter.

To further investigate whether SREBF2 mediates the downregulation of miR-184 during follicular atresia, its expression pattern was detected. Based on RNA-seq data, RT-qPCR, and western blotting analysis, we found that *SREBF2* was also significantly downregulated during follicular atresia (Fig. 7I–K), which had a similar alternative pattern to miR-184. Notably, the enrichment of SREBF2 on *miR-184* promoter in GCs from healthy follicles was higher than that from atretic follicles (Fig. 7L), indicating that restricted expression of SREBF2 lead to the downregulation of miR-184. In addition, bioinformatic analysis showed that high levels of H3K4me3 and H3K27ac, rather than CpG island, existed in the promoter of *miR-184* (Fig. S7E, F). Moreover, treatment with H3K4me3 inhibitor (BCI-121), rather than H3K27ac inhibitor (ITSA-1) or DNA methylation inhibitor (5-AzaC), dramatically suppressed *miR-184* expression in GCs (Fig. 7M). Unexpectedly, BCI-121 treatment dramatically reduced the enrichment of SREBF2 on *miR-184* promoter (Fig. 7N), but had no effect on its expression (Fig. 7O), indicating that H3K4me3-meidated alterations of chromatin opening influences the recognition and binding of SREBF2 to *miR-184* promoter. Meanwhile, comparative analysis showed that H3K4me3 enrichment on *miR-184* promoter was reduced during follicular atresia (Fig. 7P). Collectively, these findings demonstrate that restricted SREBF2 expression and inhibited H3K4me3 modification lead to the downregulation of *miR-184* in GCs during follicular atresia.

### miR-184 is essential for the development of sow follicles

**Fig. 8:**
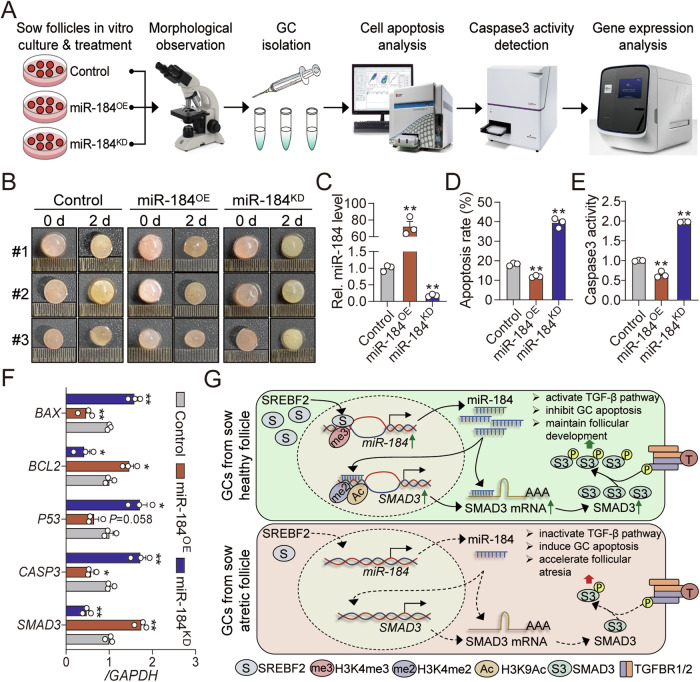
miR-184 is essential for the development of sow follicles. **A** The flow diagram showing the methodology employed in this study, which is involved in sow follicles in vitro culture system and multiple analysis. **B** Effects of miR-184 on the development of sow follicles cultured in vitro were detected by morphological analysis (*n* = 6). **C** Overexpression and knockdown efficiency of miR-184 in GCs from in vitro cultured sow follicles were analyzed using RT-qPCR (*n* = 3). **D** Effect of miR-184 on the apoptosis of GCs from in vitro cultured sow follicles was quantified by FACS (*n* = 3). **E** In vitro cultured sow follicles were treated as indicated, and Caspase3 activity in the internal GCs was detected by ELISA (*n* = 3). **F** miR-184 mimics or inhibitor was transfected into sow follicles cultured in vitro, the expression levels of apoptosis-related genes (*BAX*, *BCL2*, *P53*, and *Caspase3*) and *SMAD3* in the internal GCs were analyzed by RT-qPCR (*n* = 3). **G** Schematic diagram depicting the downregulation mechanism of miR-184 during follicular atresia, along with the working model of miR-184 inhibits GC apoptosis and follicular atresia by inducing *SMAD3* expression and TGF-β pathway activity. Data in (**C**–**F**) were shown as mean ± SEM. Significance were analyzed using ANOVA. *P < 0.05, **P < 0.01.

The apoptosis of GCs is the primary cause of follicular atresia. Therefore, we proceeded to investigate the effects of miR-184 on the development of sow follicles with a strategy based on in vitro follicle culture system and multiple methodologies, as illustrated in Fig. 8A. Morphological analysis revealed that knockdown of miR-184 accelerated the atretic process of sow follicles cultured in vitro, which was characterized by paleness, turbidity, and vascular regression (Fig. 8B, C). Conversely, overexpression of miR-184 could maintain the healthy state of follicles, indicating that miR-184 plays an important role in sow follicular development. Besides, the internal GCs were isolated and analyzed, and we noticed that inhibition of miR-184 significantly induced GC apoptosis, elevated Caspase3 activity, promoted the expression of apoptosis-related genes (*BAX*, *P53*, and *Caspase3*), and inhibited the expression of *SMAD3* (Fig. 8D–F). While, the opposite results were observed following the treatment with miR-184 mimics. Taken together, these findings demonstrate that miR-184 is essential for maintaining follicular development, alleviating GC apoptosis, and inhibiting follicular atresia in sows.

## Discussion

With the assistance of high-throughput sequencing technology, recent researches have indicated that numerous miRNAs potentially affect follicular atresia [32]. Thus, identifying effective miRNAs is essential for follicular atresia inhibition, ovulation increasing, female reproduction maintenance, and animal husbandry improvement. Differentially expressed genes from omics analysis are mainly screened based on expression alteration and statistical significance, but their fundamental levels and tissue or cell specificity are often overlooked, which is crucial for functional assessment [33]. The specific high expression of a miRNA indicates that it maintains the characteristics and basic functions of the tissue/cell, whereas downregulation of its expression during a physiological or pathological process implies its inhibitory effect in this process, and vice versa. For instance, miR-141 was highly expressed in human and mouse airway epithelium, and was altered in bronchial brushings during asthma, further confirming that it regulates mucus production [34]. Besides, high expression level of miR-451 is inverted in the follicular fluid of women with endometriosis, which may serve as a biomarker of endometriosis and embryo quality [35]. Currently, more attention is paid to identify the miRNAs with low basic expression levels but significantly upregulated during follicular atresia in the ovary of female mammals, which might have pro-atretic effects by inducing GC apoptosis [25, 36, 37]. Only a few studies have examined the anti-atretic effects of downregulated OE miRNAs [38], and the underlying mechanisms remain largely unknown. In 2019, Chen et al. established global miRNA tissue expression profile in pig through RNA-seq [39]. Based on which, we systematically screened the downregulated OE miRNAs in sow GCs for the first time by combining with our previous comparative high-throughput data. Following analysis, the abundance of three miRNAs (miR-184, miR-210, and miR-371) were effective to predict and diagnose follicular atresia in sows, which provides a theoretical basis for the development of non-hormonal endogenous regulators to inhibit follicular atresia.

In accordance with tissue expression characteristics, previous investigation into miR-184 have indicated that it is associated with cardiac differentiation and damage, adaptive immune response, organ metabolism, adipogenesis, neurodegenerative disease, and carcinogenesis [40–46]. Despite its specific high expression in ovary tissue, only a few studies have focused on the functions of miR-184 in the female reproductive system. In 2009, Iovino et al. first highlighted the importance of miR-184-mediated female germline development [47]. Zhang et al. reported that miR-184 regulated spontaneous abortion through promoting trophoblast cell apoptosis [48]. Besides, low miR-184 abundance was found correlated with tumor recurrence in ovarian GC tumor patients [49]. In this study, miR-184, a downregulated OE miRNA, was selected as a subject after comprehensive analyses of expression alteration, chromosome location, and ROC diagnosis. Further functional evaluation with in vitro GC and follicle culture system demonstrated that miR-184 exerts anti-atretic effects by inducing the proliferation and inhibiting the apoptosis of GCs. Combined with the findings of Shi et al. that miR-184 promoted estradiol synthesis in GCs [50] indicates that miR-184 is essential for maintaining the normal state and function of GCs. In addition, miR-184 was found highly expressed in GCs from dominant follicles in cows [51], and involved in the phthalates-mediated oocyte maturation in rats [52]. Collectively, these findings indicate that miR-184 is a pivotal regulator for follicular development in female mammals. It is noteworthy that downregulated OE miRNAs (including miR-184) are located in the pig reproduction traits-associated QTLs, which may partially explain their high expression levels in ovary tissue of high-yielding breeds or individuals [50, 53].

It is well-known that the interactions between TGF-β pathway and miRNAs are involved in multiple crucial biological processes [54, 55]. Briefly, members of TGF-β pathway are targeted and inhibited by at least one miRNA, while the biogenesis of miRNAs is also regulated by TGF-β directly or via SMADs with their transcription factor activity [56]. In ovary tissue, TGF-β1 has been reported to influence the stability of miRNA transcriptome [57, 58], and to induce the binding of nc886 to Dicer, which inhibits miRNAs maturation and leads to ovarian cancer [59]. Conversely, miRNAs such as miR-126, miR-130a, and miR-26b inhibit the activity of TGF-β pathway by directly targeting its ligands, receptors, and SMADs, further promote GC apoptosis, induce follicular atresia, and impair fertility [15, 60, 61]. Here, we demonstrated that miR-184 induces *SMAD3* transcription by acting as a saRNA, and maintains the stability of SMAD3 mRNA by binding to its 5′-UTR, which further activates TGF-β pathway and inhibits sow GC apoptosis. To our knowledge, this is the first instance of direct interaction between miRNA and SMAD3 in ovary of female mammals, which is obviously distinct from the cases where miRNAs inhibit *SMAD3* expression via canonical inhibitory mechanism in other tissues [62]. Similar to other non-coding RNAs, the regulatory mechanism of miRNAs is also determined by their subcellular localization. Specifically, miRNAs in the nucleus induce the transcription of target genes by binding to their promoters and altering the histone modification-mediated chromatin opening, which is also known as saRNA mechanism [63]. In addition to the classical mechanism, cytoplasmic miRNAs are able to regulate the stability of target mRNAs by directly binding to their 5′-UTR, but the regulatory effect is bidirectional [38, 64]. Li et al. reported that binding of miRNAs to structured 5′-UTR could elevate the stability of target mRNAs, while accelerates mRNA degradation when binding to linear 5′-UTR [65]. Interestingly, secondary structure analysis reveals that the 5′-UTR of SMAD3 mRNA is highly structured (Fig. S9), which partially explains our findings.

The biogenesis of mature miRNAs is a multi-step process that involves transcription, trimming, exporting, and cleavage [66]. Presently, the post-transcriptional processing of miRNAs is well-defined dependent on the Dgcr8/Drosha-Exportin5-Dicer system. However, the regulatory mechanism of miRNA transcription is vague and not fully understood, mainly due to two reasons: (1) host genes, and (2) complicated transcriptional regulation patterns. A notable finding is the high prevalence (>60%) of miRNAs with host genes, mostly located on the sense strand (>80%) and predominantly mapping into introns (~90%) [67]. For intergenic miRNAs, their transcription is explicitly independent. While, examples of coordinated and independent expression of intragenic miRNAs and their host genes have been reported [68, 69]. Franca et al. proposed that host gene constraints and genomic context impacts the expression of miRNAs, and sense intronic miRNAs with the same transcription direction tend to be co-transcribed with host genes [70]. Here, we found that *miR-184*, a sense intragenic miRNA located in the first intron of *LOC10657693*, is independently transcribed, which is similar to miR-425 identified in our previous study [37], indicating that the coordinated transcription mechanism of miRNAs and their host genes remain to be fully elucidated. In addition, recent studies demonstrated that miRNAs are also regulated by TFs and chromatin accessibility, consistent with protein-coding genes [71, 72]. Here, we confirmed that miR-184 is transcriptionally activated in sow GCs by SREBF2, a TF associated with follicular development and E2 synthesis [73, 74], in an H3K4me3-dependent manner, which reveals the specific high expression of miR-184 in the ovary and its downregulation pattern during follicular atresia. Dynamic alteration in chromatin accessibility is the primary cause of instability in transcriptome during biological processes, including follicular development [75], which may be explained by (1) histone modification system disorder, (2) cell state and micro-environment alteration, (3) hormone, cytokine, and signals stimulation. Therefore, further investigations are still required to uncover the underlying mechanism of chromatin accessibility changes in GCs during follicular atresia.

Unlike other non-coding RNAs, miRNAs among species are deeply conserved [76], so as the miR-184. It is also noteworthy that the homology and consistency of miR-184 were found throughout this study, including not only sequence, chromosome localization, host and neighbour genes. Functionally, miR-184 exerts anti-apoptotic effects in both sow and human GCs through promoting *SMAD3* expression with the same mechanism (acts as a saRNA and stabilizes mRNA), and its responsive elements among mammals are highly conserved. More importantly, the expression patterns of miR-184 during follicular atresia are consistent across mammals, while the binding site of SREBF2 as well as the histone modification within the promoter of *miR-184* exhibit high levels of consistency. These results indicate that the downregulation patterns, effector targets, biological functions, and regulatory mechanisms of miR-184 are highly conserved and universal. Further studies should clarify its regulatory roles in female fecundity via in vivo experiments with human and model animals.

In summary, we systematically identified the downregulated OE miRNAs and revealed their anti-atretic effects in sow follicles. miR-184, a notable conserved downregulated OE miRNA, inhibited GC apoptosis and follicular atresia through inducing *SMAD3* expression and elevating TGF-β pathway activity by acting as a saRNA and mRNA stabilizer. Furthermore, the downregulation mechanism of miR-184 during follicular atresia was elucidated, which was regulated by SREBF2 in an H3K4me3-dependent manner. These findings highlight the crucial roles of downregulated OE miRNAs in the regulation of GC apoptosis and follicular atresia, and contribute to the development of endogenous non-hormonal regulators for improving follicular development, ovarian health, and female fertility.

## Supplementary information

Supplementary Tables

Supplementary Figures

Full and uncropped western blots and gel images

## Data Availability

All data generated during this study are included in this published article and its Supplementary Information files.
