# Supplementary material for: miR-184, a downregulated ovary-elevated miRNA transcriptionally activated by SREBF2, exerts anti-apoptotic properties in ovarian granulosa cells through inducing SMAD3 expression
Source: Cell Death Dis. 2024 Dec 18;15(12):892. doi: 10.1038/s41419-024-07286-1 (PMC11655972; doi:10.1038/s41419-024-07286-1)
Supplement: Supplementary file 2 — Supplementary Figures [file 41419_2024_7286_MOESM2_ESM.docx]

**miR-184, a downregulated ovary-elevated miRNA transcriptionally activated by SREBF2, exerts anti-apoptotic properties in ovarian granulosa cells through inducing *SMAD3* expression**

Baosen Shan^1^, Yangan Huo^1^, Zhennan Guo^1^, Qiqi Li^1, 2^, Zengxiang Pan^1^, Qifa Li^1^ and Xing Du^1,*^

1 College of Animal Science and Technology, Nanjing Agricultural University, Nanjing 210095, China.

2 College of Animal Husbandry and Veterinary Medicine, Jiangsu Vocational College of Agriculture and Forestry, Zhenjiang 212400, China.

* Corresponding author: Xing Du (duxing@njau.edu.cn).

**Supplementary Figures**


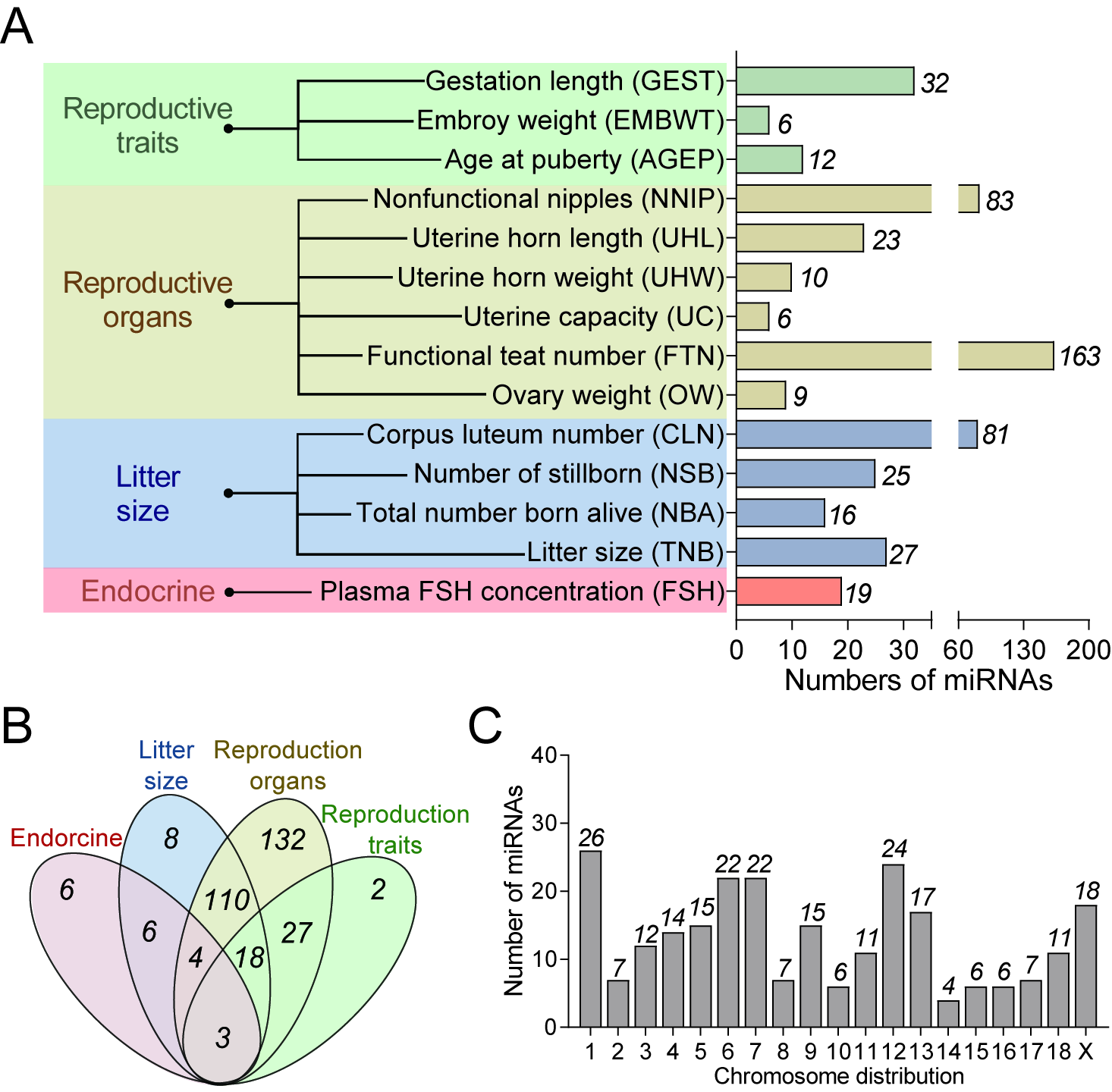


**Fig. S1 Global identification of the miRNAs located in the QTLs associated with reproduction traits in pig**. **A** Statistics analysis of the miRNAs in different QTL categories of pig reproduction traits. **B** Venn diagram showing the common miRNAs in multiple QTL categories of pig reproduction traits. **C** Chromosome distribution of the pig reproduction trait-associated miRNAs. The numbers in **A-C** indicate the amounts of miRNAs located within pig reproduction trait-associated QTLs.

**
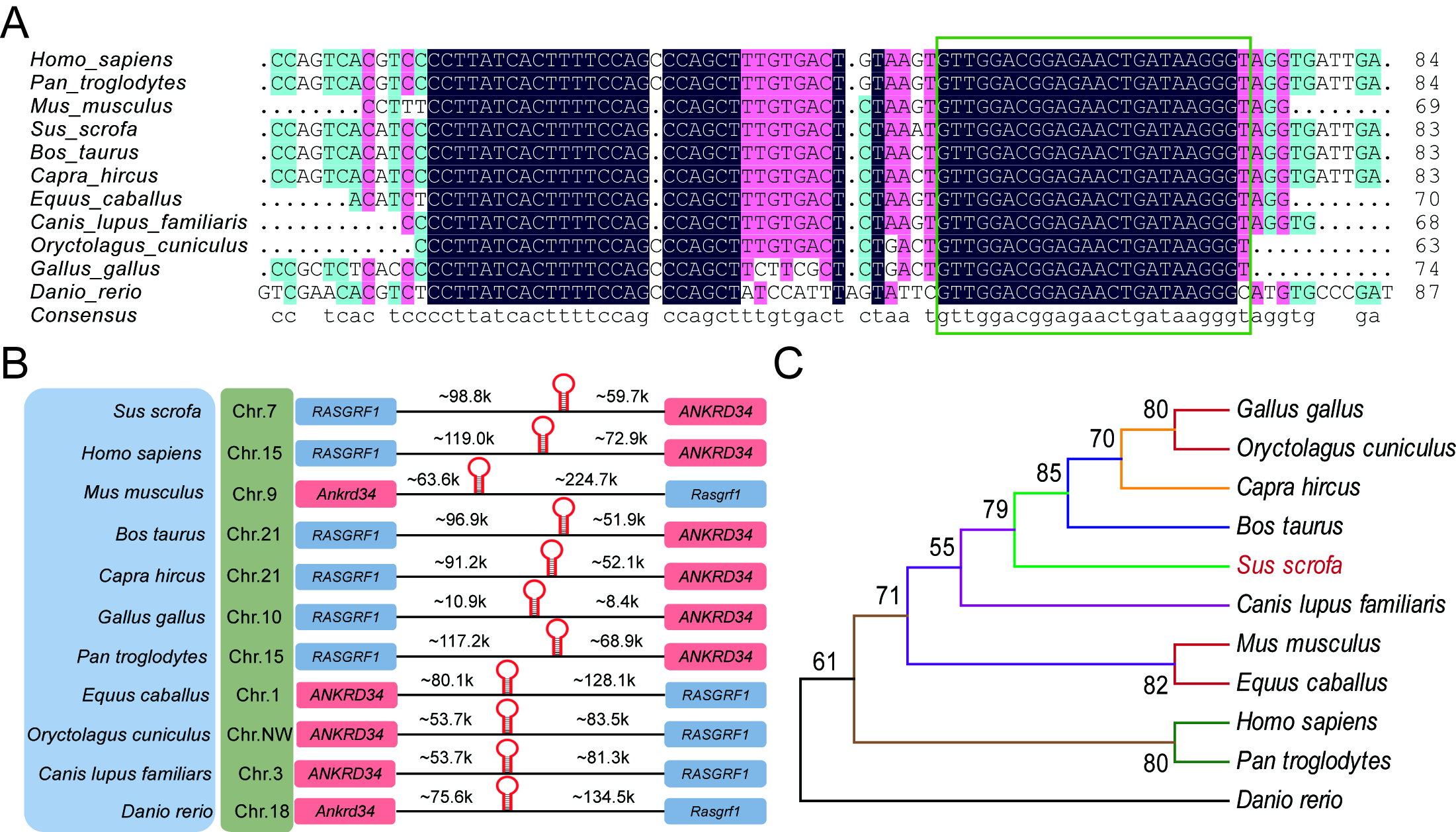
**

**Fig. S2 miR-184 is a highly conserved miRNA among multiple species. A** Alignment analysis of pre-miR-184 nucleotide sequences from 11 different species. Mature sequences of miR-184 were indicated with green box. **B** Chromosome locations of miR-184 from different species and their neighboring protein-coding genes were analyzed and visualized. The red stem-loop structure indicated miR-184. **C** Evolutionary conservation analysis of miR-184 among different species.


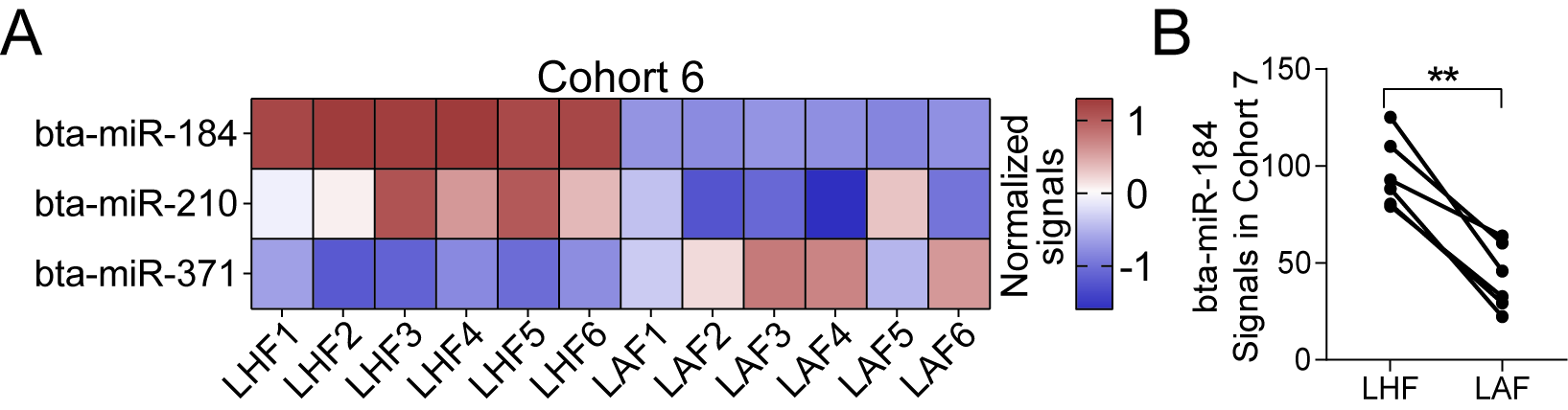


**Fig. S3 miR-184 is dramatically downregulated in bovine follicles during atresia. A** Heatmap depicting the normalized signals of miR-184, miR-210, and miR-371 in bovine large healthy follicles (LHF, n=6) and large atretic follicles (LAF, n=6) from Cohort 6 (GSE54692, microarray). **B** The expression alteration pattern of bta-miR-184 in six pairs of bovine LHFs and LAFs from Cohort 7 (GSE87347, microarray). ** *P* < 0.01.


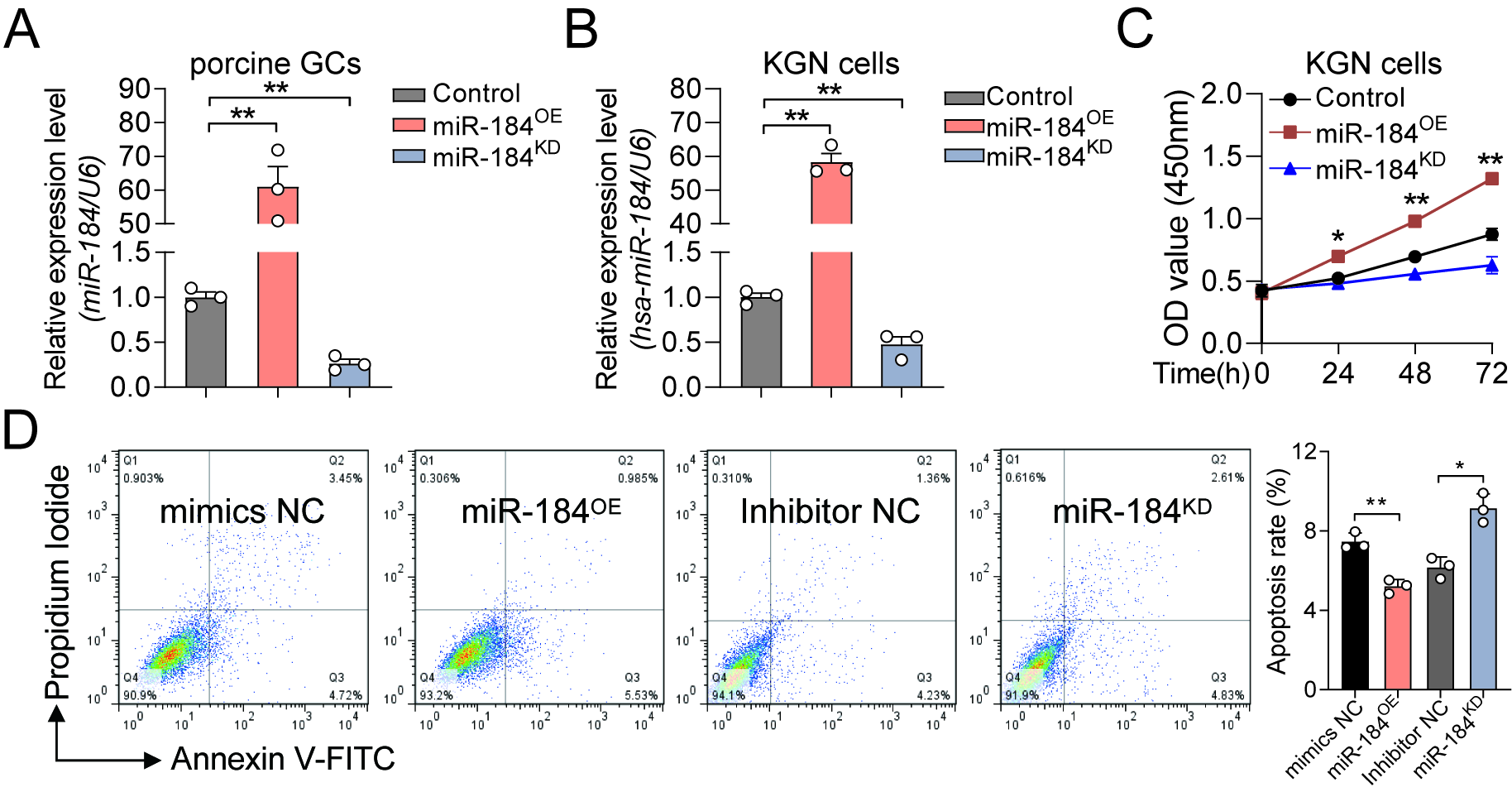


**Fig. S4 miR-184 exerts proliferative and anti-apoptotic functions in KGN cells. A** miR-184 expression levels in porcine GCs transfected with miR-184 mimics or inhibitors were analyzed using RT-qPCR (n=3). **B** RT-qPCR was performed to detect the over-expression and inhibition efficiency of miR-184 in KGN cells (n=3). **C**-**D** Effects of miR-184 on the proliferation and apoptosis of KGN cells were detected by CCK-8 (**C**) and FACS (**D**) assays. Data were shown as mean ± SEM with at least three independent replicates. Significance were calculated using two-tailed student’s *t*-test. * *P* < 0.01, ** *P* < 0.01.


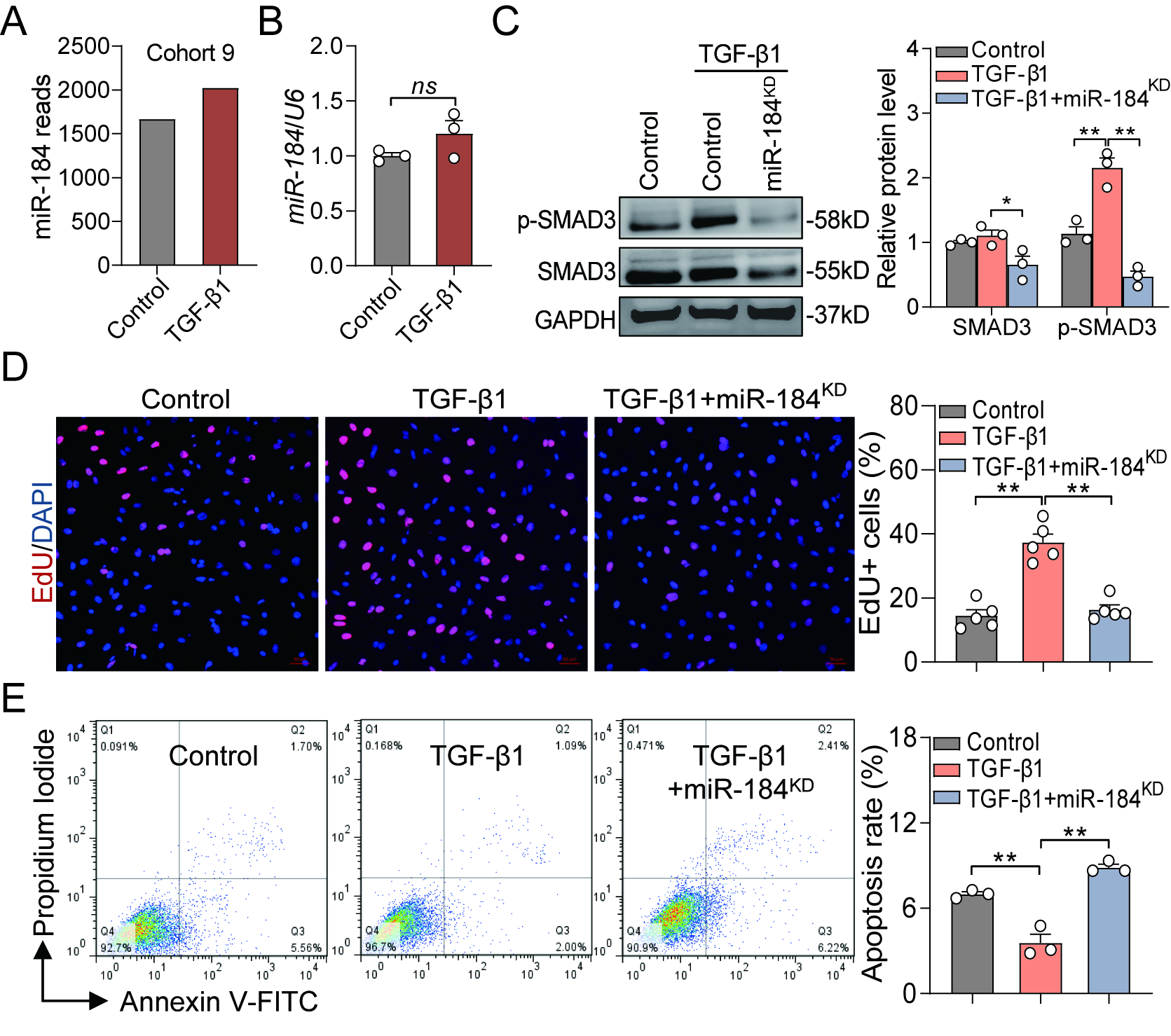


**Fig. S5 Knockdown of miR-184 impairs the biological functions of TGF-β1 in sow GCs. A** miR-184 reads in sow GCs treated with 10 ng/mL TGF-β1 were obtained from previous RNA-seq data (Cohort 10). **B** Effect of 10 ng/mL TGF-β1 on miR-184 expression in sow GCs was detected using RT-qPCR (n=3). **C**-**E** Sow GCs were co-treated with 10 ng/mL TGF-β1 and miR-184 inhibitor for 48h, SMAD3 and p-SMAD3 protein levels were measured using western blotting (**C**), cell proliferation was detected by EdU staining (**D**), and cell apoptosis was analyzed by FACS (**E**). Data were shown as mean ± SEM with at least three independent replicates. Significance were analyzed by two-tailed student’s *t*-test and ANOVA. * *P* < 0.01, ** *P* < 0.01, and *ns* indicates no significance.


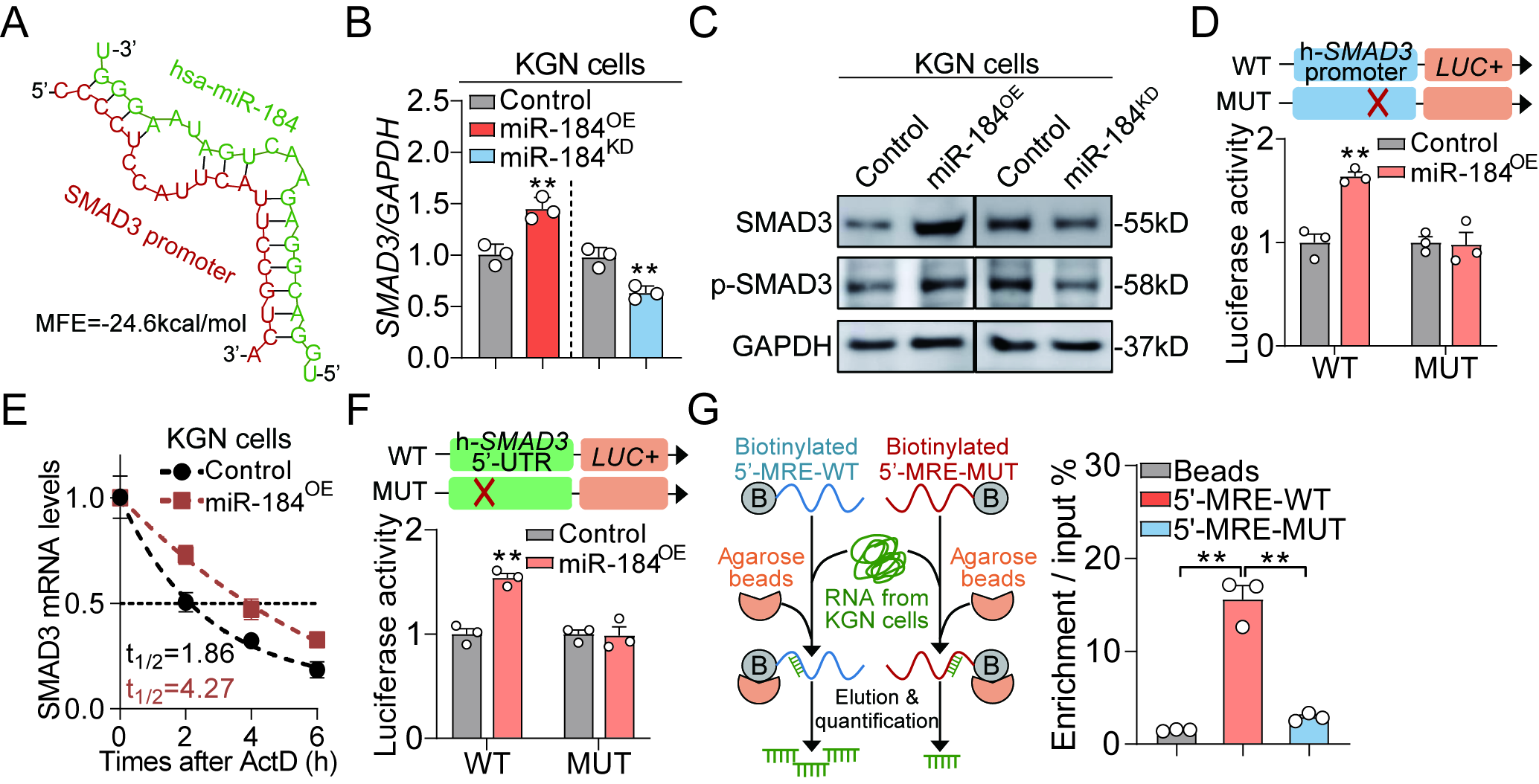


**Fig. S6 miR-184 induces *SMAD3* expression in KGN cells through a universal regulatory mechanism. A** Hsa-miR-184 response element (MRE) on the promoter of human *SMAD3* was analyzed using RNAhybrid software, and the minimal free energy (MFE) was calculated. **B, C** The effects of hsa-miR-184 on the mRNA (**B**) and protein (**C**) levels of SMAD3 in KGN cells were measured using RT-qPCR and western blotting assays (n=3). **D** Dual-luciferase reporter assay was conducted to analyze the effects of hsa-miR-184 on the activity of vectors containing human *SMAD3* promoter with wild-type (WT) or mutant (MUT) MRE (n=3). **E** The effects of hsa-miR-184 on the stability of SMAD3 mRNA in KGN cells were detected using ActD chase assay. **F** The influence of hsa-miR-184 on the luciferase activities of 5’-UTR of human SMAD3 mRNA with the wild-type (WT) or mutant (MUT) MRE (n=3). **G** The physical binding and interaction region between 5’-UTR of human *SMAD3* and hsa-miR-184 were detected using in vitro RNA pull-down assay. Data were shown as mean ± SEM with at least three independent replicates. Significance were analyzed by two-tailed student’s *t*-test. ** *P* < 0.01.

**
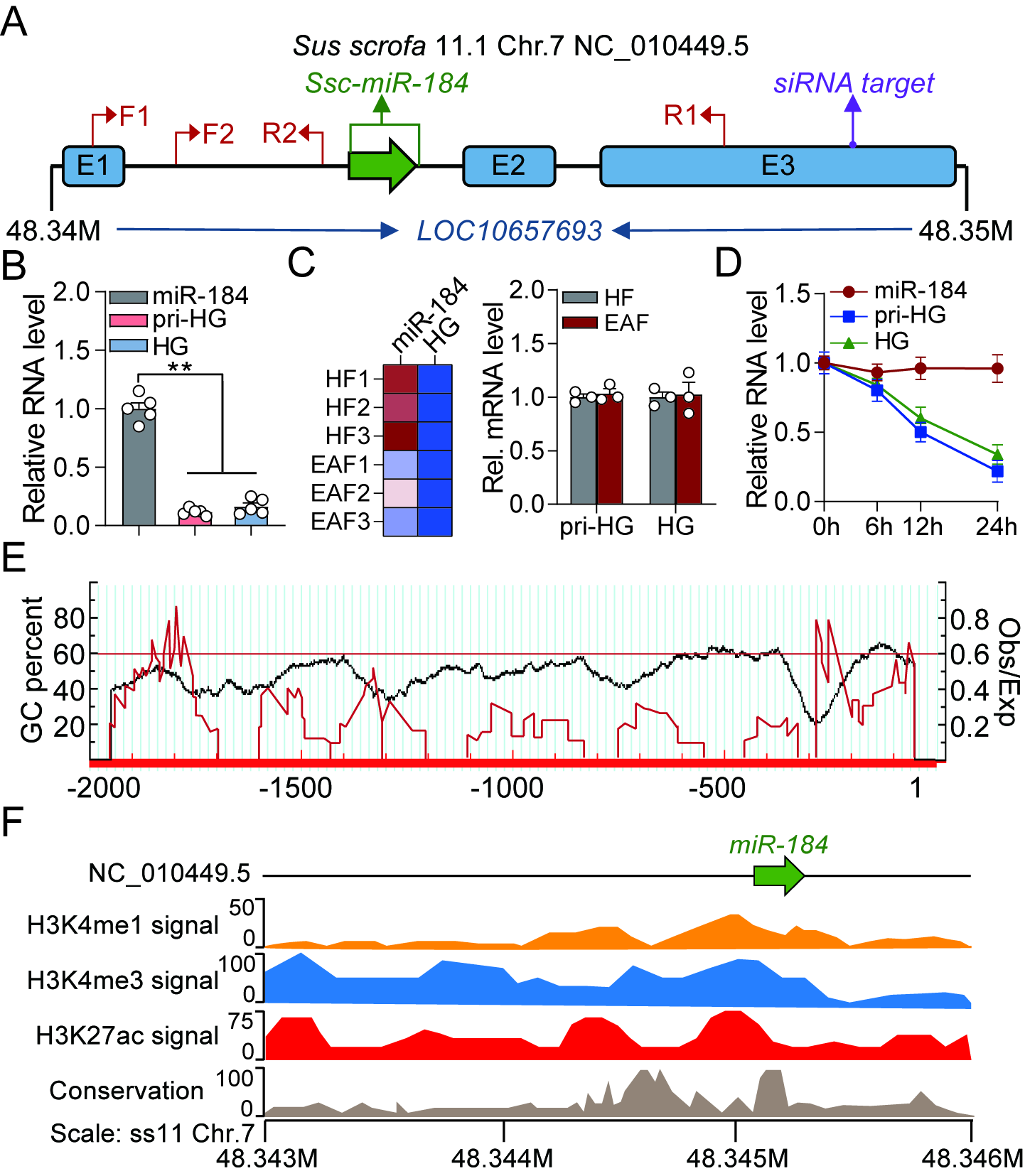
**

**Fig. S7 *Ssc-miR-184* is transcribed independently of its host gene *LOC106507693*. A** Diagram showing the chromosome location of *ssc-miR-184* (green) and its host gene *LOC106507693* (blue). F1/R1 and F2/R2 indicate primer pairs used to detect the expression level of mature LOC106507693 (HG) and pri-LOC106507693 (pri-HG), respectively. siRNA-target indicates the designed siRNA specifically for pri-HG inhibition. **B** The expression levels of miR-184, pri-HG, and HG in sow GCs were detected using RT-qPCR (n=5). **C** The alteration of pri-HG and HG in GCs during follicular atresia was analyzed by RNA-seq (left panel) and RT-qPCR (right panel, n=3). **D** The expression levels of miR-184, pri-HG, and HG in GCs after transfected with pri-HG siRNA for indicated times were analyzed using RT-qPCR. **E** GC percent (black) and potential CpG island (red) within *ssc-miR-184* promoter were analyzed by Methprimer and EBI CpGplot. **F** Chromatin states of ssc-miR-184 and its promoter region were obtained from the UCSC Genome Browser database, including histone modification and conservation. Data were shown as mean ± SEM with at least three independent replicates. ** *P* < 0.01.


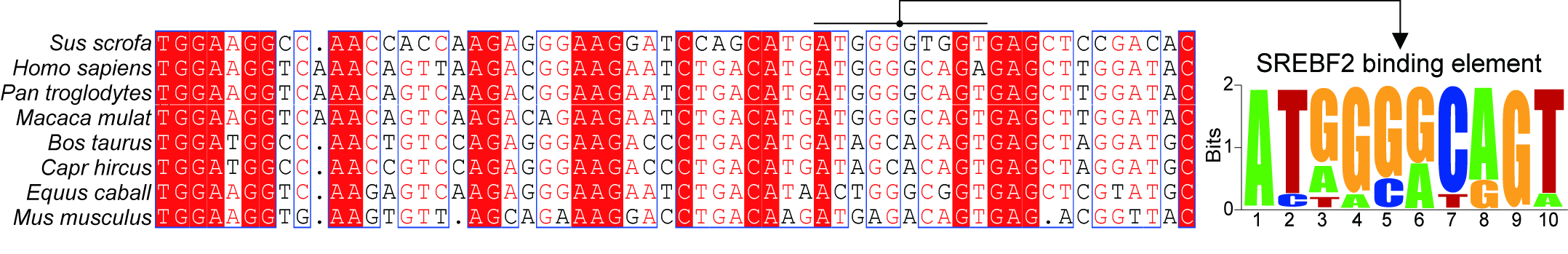


**Fig. S8 Sequence alignment of SREBF2 binding element within the promoter of *miR-184* among different mammals.** The responsive elements of SREBF2 within the promoter of *miR-184* are highly conserved among different mammals.


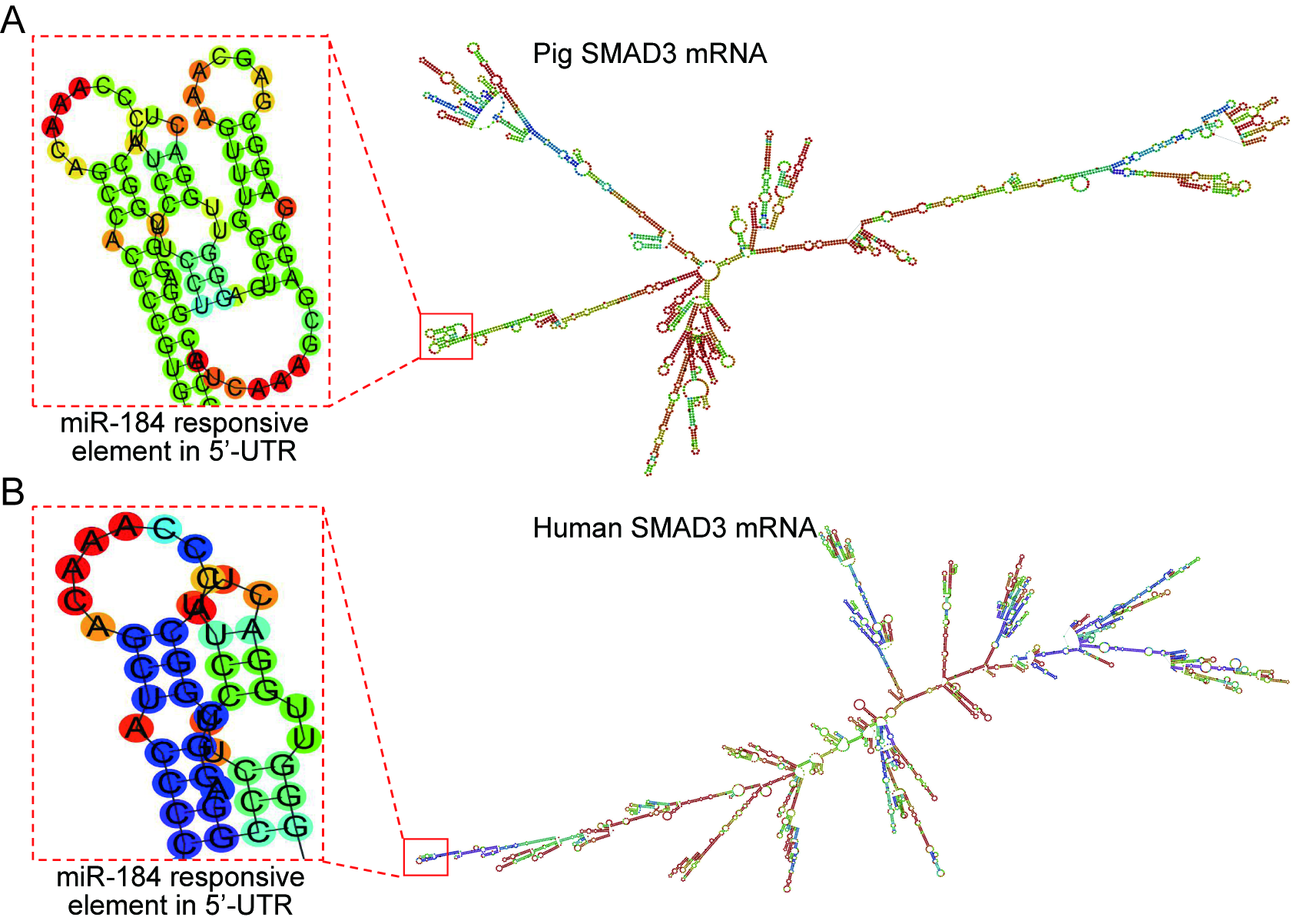


**Fig. S9 miR-184 responsive elements in the 5’-UTR of SMAD3 mRNA are highly structured. A, B** The secondary structure of SMAD3 mRNA from pig (**A**) and human (**B**) were analyzed by Mfold software, and the responsive elements of miR-184 in their 5’-UTR were enlarged and represented with red dotted boxes, which are highly structured.
