# Supplementary material for: miR-184, a downregulated ovary-elevated miRNA transcriptionally activated by SREBF2, exerts anti-apoptotic properties in ovarian granulosa cells through inducing SMAD3 expression
Source: Cell Death Dis. 2024 Dec 18;15(12):892. doi: 10.1038/s41419-024-07286-1 (PMC11655972; doi:10.1038/s41419-024-07286-1)

Original blot images

Figure 4F

SMAD3

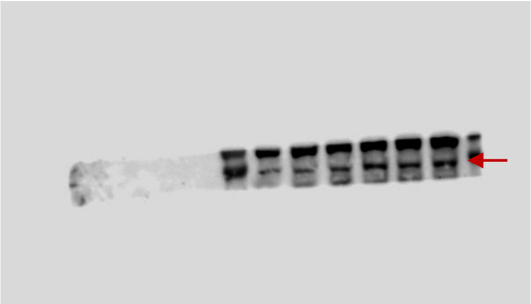

p-SMAD3

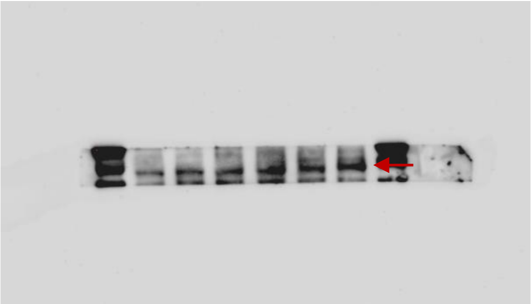

TGF-β1

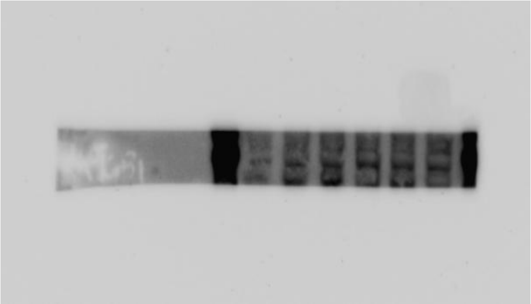

TGFBR2

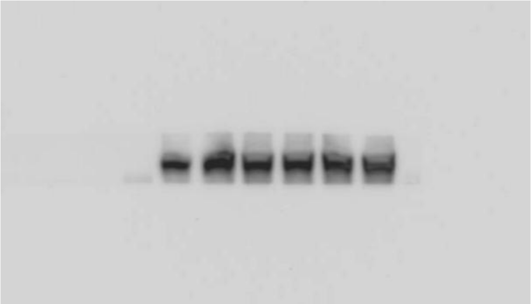

SMAD4

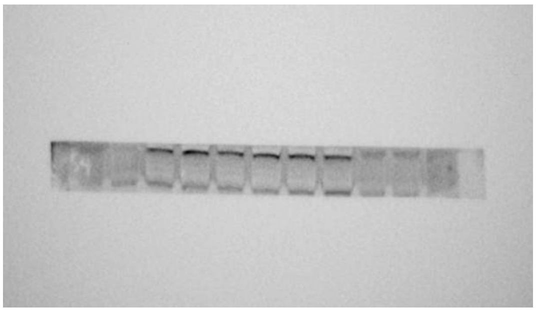

SMAD7

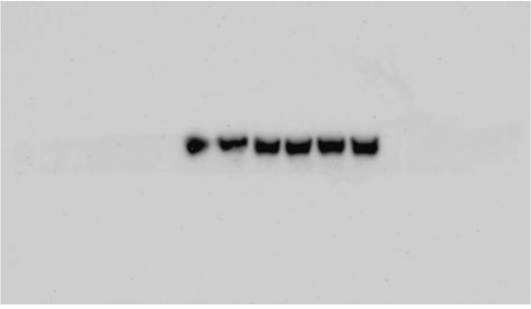

GAPDH

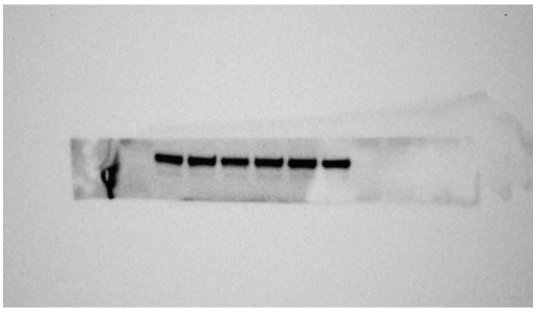

Figure 4G

SMAD3

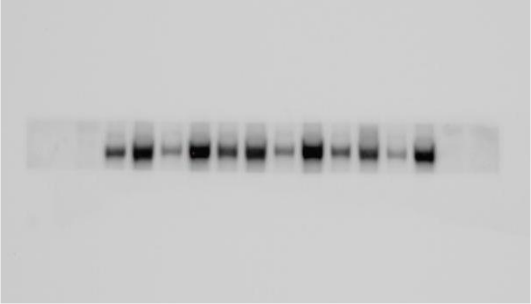

p-SMAD3

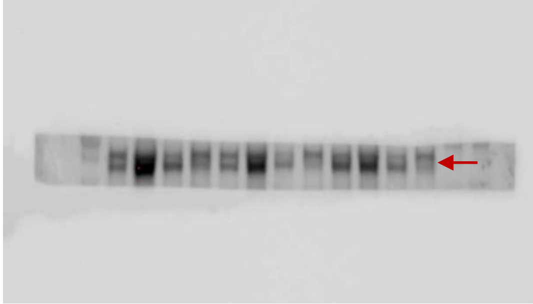

GAPDH

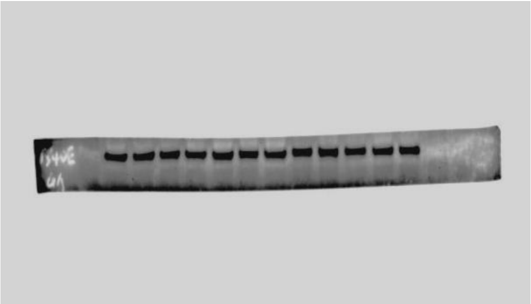

Figure 6C

AGO2

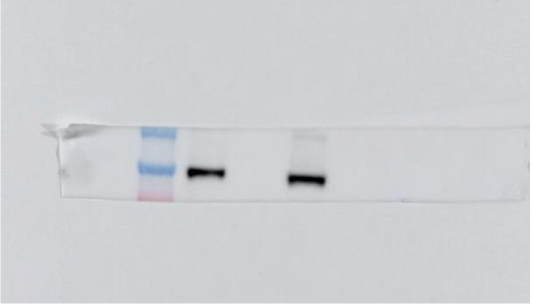

Figure 7K

SREBF2

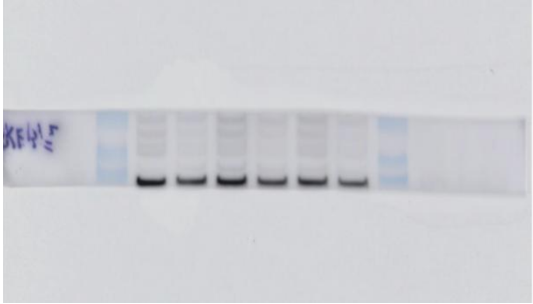

GAPDH

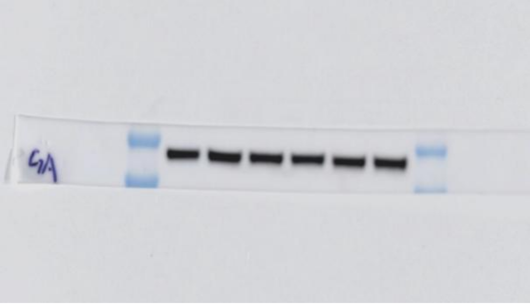

Figure S5C

SMAD3

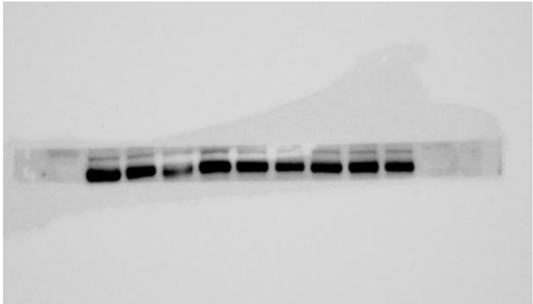

p-SMAD3

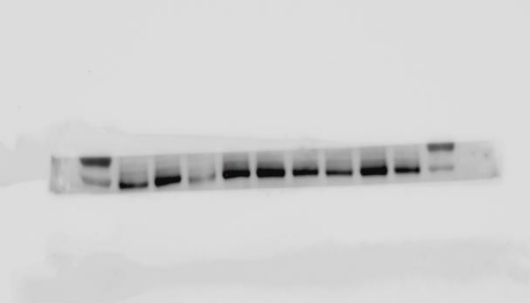

GAPDH

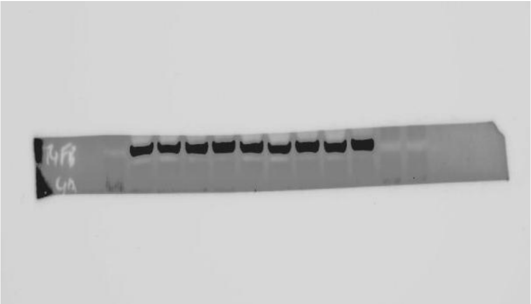

Figure S6C

SMAD3-left

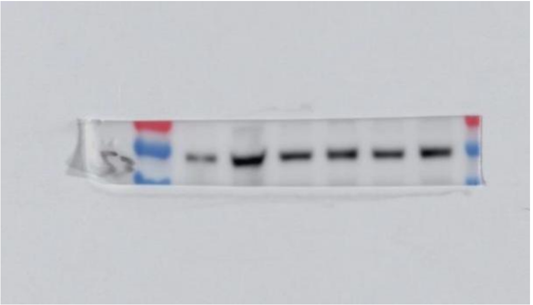

SMAD3-right

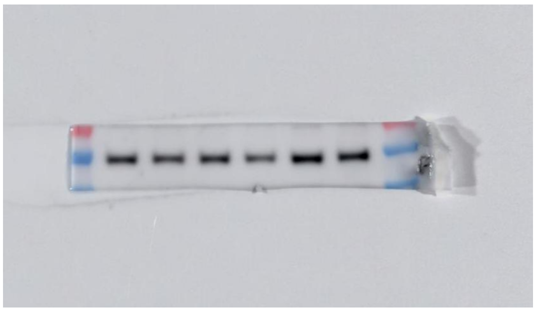

p-SMAD3-left

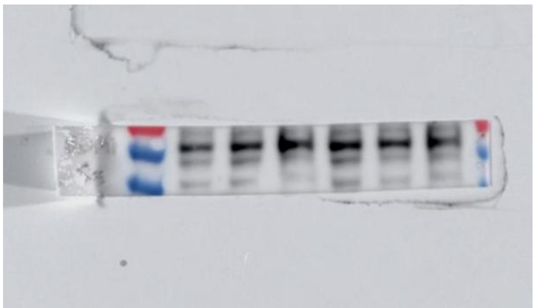

p-SMAD3-right

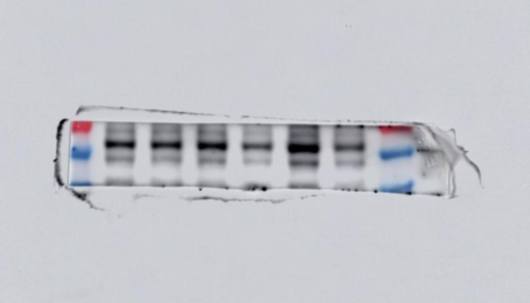

GAPDH-left

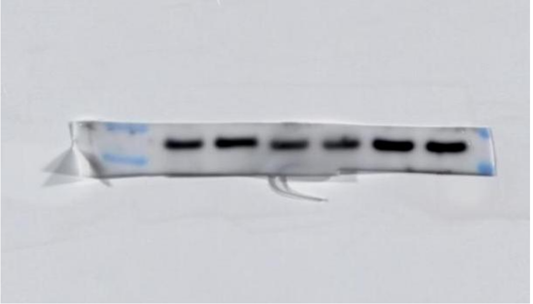

GAPDH-right

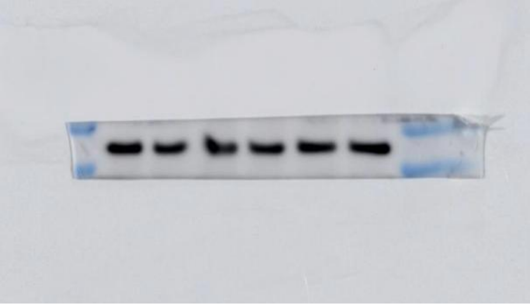

Original gel images

Figure 5C

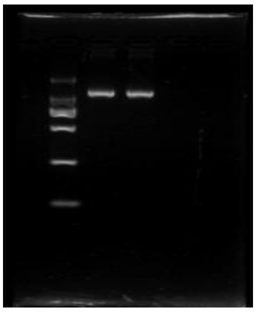

Figure 5G F1/R1

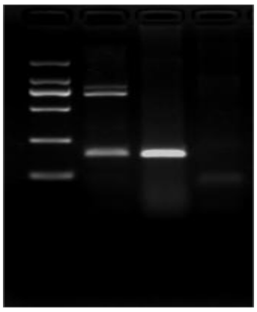

F2/R2

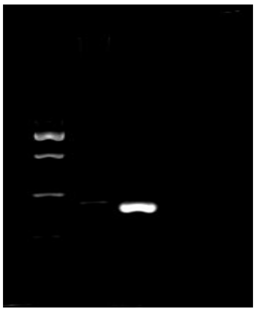

F3/R3

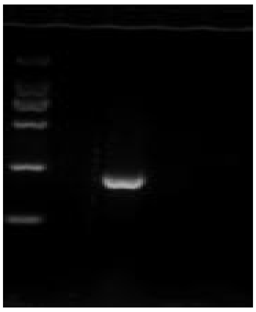

Figure 5I

F1/R1-H3K9Ac

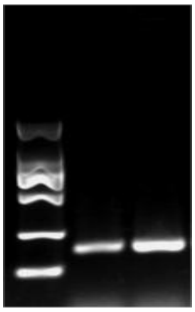

F1/R1-H3K4me2

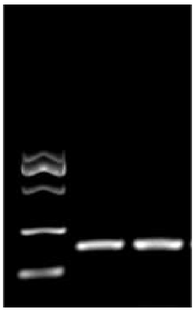

F1/R1-Input

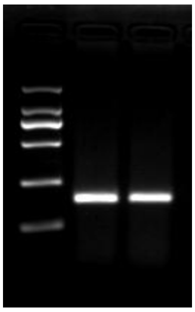

F2/R2-H3K9Ac

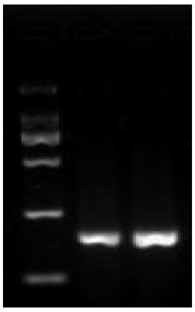

F2/R2-H3K4me2

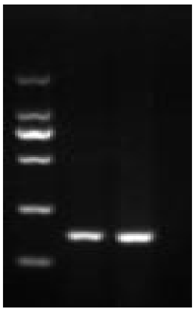

F2/R2-Input

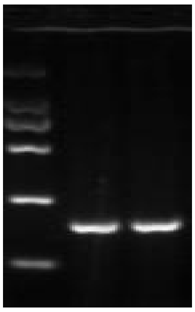

F3/R3-H3K9Ac

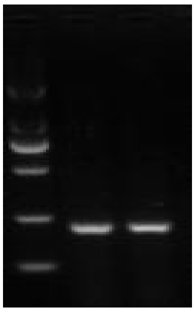

F3/R3-H3K4me2

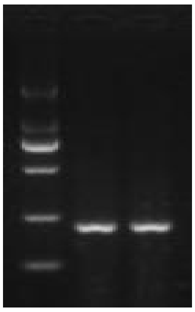

F3/R3-Input

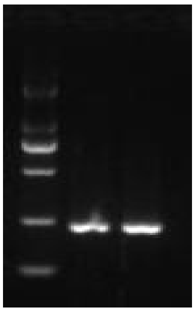

Figure 6C

miR-184

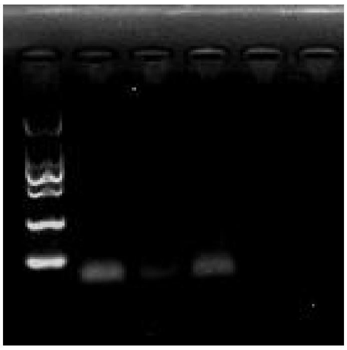

SMAD3 mRNA

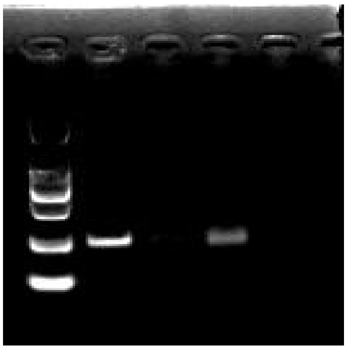

Figure 6D

SMAD3 mRNA

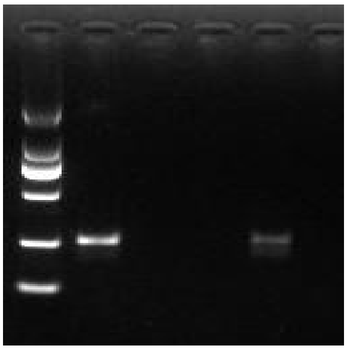

Figure 7G

SBE

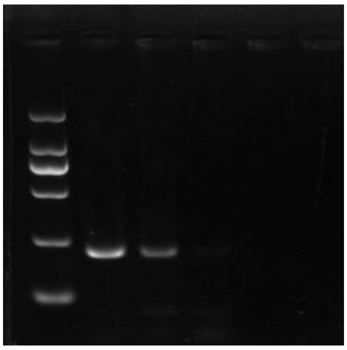

SBEX

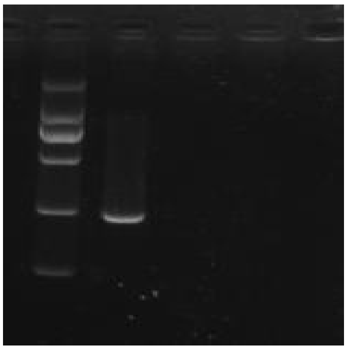

Figure 7L

SBE

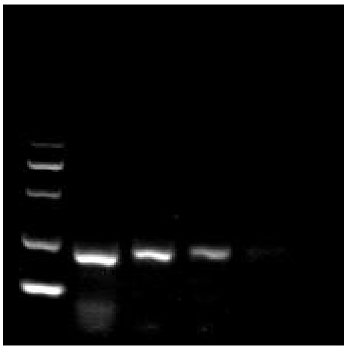

Figure 7N

SBE-upper

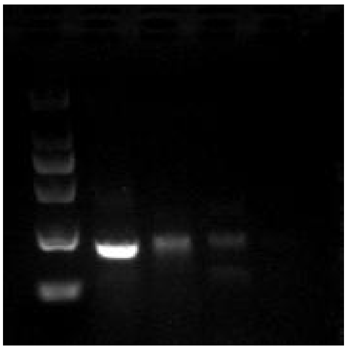

SBEX-lower

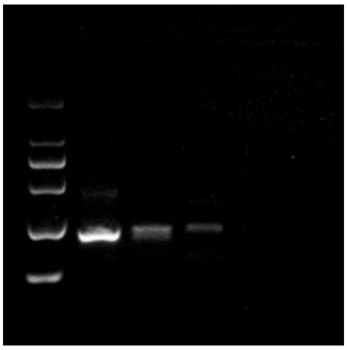

Figure 7P

SBE

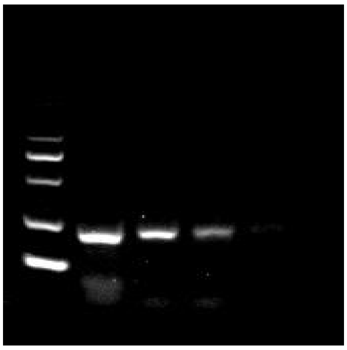

Supplement: Supplementary file 3 — Full and uncropped western blots and gel images [file 41419_2024_7286_MOESM3_ESM.pdf]
